# Circadian Clock Regulates Inflammation and the Development of Neurodegeneration

**DOI:** 10.3389/fcimb.2021.696554

**Published:** 2021-09-14

**Authors:** Xiao-Lan Wang, Lianjian Li

**Affiliations:** ^1^Department of Nephrology, Union Hospital, Tongji Medical College, Huazhong University of Science and Technology, Wuhan, China; ^2^Department of Surgery, Hubei Provincial Hospital of Traditional Chinese Medicine, Wuhan, China; ^3^Hubei Province Academy of Traditional Chinese Medicine, Wuhan, China

**Keywords:** circadian clock, immune response, systemic inflammation, neurodegeneration, cellular metabolism

## Abstract

The circadian clock regulates numerous key physiological processes and maintains cellular, tissue, and systemic homeostasis. Disruption of circadian clock machinery influences key activities involved in immune response and brain function. Moreover, Immune activation has been closely linked to neurodegeneration. Here, we review the molecular clock machinery and the diurnal variation of immune activity. We summarize the circadian control of immunity in both central and peripheral immune cells, as well as the circadian regulation of brain cells that are implicated in neurodegeneration. We explore the important role of systemic inflammation on neurodegeneration. The circadian clock modulates cellular metabolism, which could be a mechanism underlying circadian control. We also discuss the circadian interventions implicated in inflammation and neurodegeneration. Targeting circadian clocks could be a potential strategy for the prevention and treatment of inflammation and neurodegenerative diseases.

## Introduction

Virtually all life has an intrinsic timing system, the so-called biological clock, which coordinates biological processes to adapt to the regular 24-hour light/dark cycles generated by the Earth’s rotation (Bell-Pedersen et al., 2005). In mammals, numerous physiological and behavioral processes exhibit a daily rhythm of approximately 24-hour, such as sleep-wake cycle, locomotor activity, feeding, body temperature, metabolism, immune response, hormone secretion, and cognition (Gerstner and Yin, 2010; Mohawk et al., 2012; Scheiermann et al., 2013; Gnocchi and Bruscalupi, 2017; Mure et al., 2018). These rhythmic activities are driven by cell-autonomous circadian clocks that existed in almost every cell in organisms. However, circadian rhythm can also be affected by external cues and pathological conditions, such as light, diet, feeding pattern, temperature, exercise, oxygen, as well as cancer, cardiovascular disease, inflammation, and neurodegenerative diseases (Cavadini et al., 2007; Kohsaka et al., 2007; Pivovarova et al., 2015; Adamovich et al., 2017; Manoogian and Panda, 2017; Hood and Amir, 2017a; Hood and Amir, 2017b; Crosby et al., 2019; Gabriel and Zierath, 2019).

The circadian system regulates numerous cellular processes, which interact and affect biological activities. For example, circadian clocks modulate cellular metabolism, epigenetic modification, cell cycle, redox homeostasis, gut microbiota, immune response, and cognition. Cellular metabolism is associated with immune activity and neurodegeneration (Camandola and Mattson, 2017; Geltink et al., 2018; Vijayan et al., 2019; Wang et al., 2019). Additionally, both central and peripheral inflammation implicate in neurodegeneration (Trager and Tabrizi, 2013; Chitnis and Weiner, 2017). Disruption of circadian rhythms leads to obesity, diabetes, cancer, immune dysfunction, and cognitive decline. Just as circadian disruption can induce biological disorders, circadian disturbances have also been regarded as the consequences of microbial infection, metabolic disorder, and neurodegenerative diseases. For example, circadian abnormality in sleep/wake cycles is one of the common and earliest signs of neurodegenerative diseases, such as Alzheimer’s disease (AD) and Parkinson’s disease (PD); while the abnormality of circadian rhythms exacerbates the progression of neurodegeneration (Hood and Amir, 2017a). Here, we review the complex circadian network and summarize the circadian control of immune function and neurodegeneration. We explore the complex interplay between systemic inflammation and neurodegeneration and discuss the potential mechanism underlying circadian regulation.

## The Circadian Clock in Mammals

**Figure 1:**
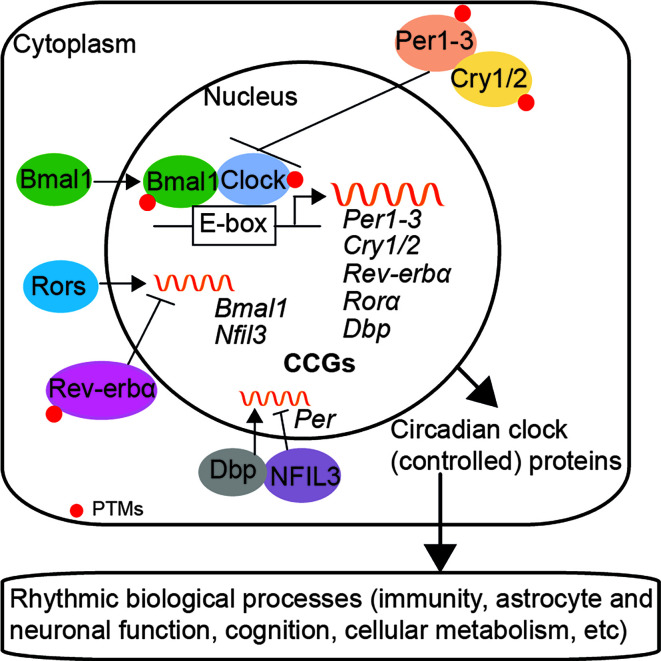
The circadian clock machinery in mammals. The mammalian circadian clock network at the molecular level consists of transcription-translation feedback loops (TTFLs). Bmal1/Clock heterodimer activates the transcription of genes that containing E-box elements in their promoters, such as *Per1/2/3*, *Cry1/2*, *Ror*α, *Rev-erb*α, and *Dbp.* Per and Cry proteins translocate back into the nucleus and repress the activity of the Bmal1/Clock complex, which subsequently inhibits their own expression. Rorα and Rev-erbα fine-tune the expression of *Bmal1* and *Nfil3*, *via* activation and repression, respectively. DBP and NFIL3 regulate the expression of *Per.* Besides, the stability and nuclear translocation of circadian clock proteins are modulated by post-translational modifications (PTMs). Circadian clocks also control the rhythmic expression of numerous clock-controlled genes (CCGs) and biological processes, such as immunity and brain function.

The circadian system is an internal timekeeping device, which is hierarchical and organized in multiple oscillators at the organism, cellular, and molecular level in mammals (Reppert and Weaver, 2002; Bell-Pedersen et al., 2005; Saini et al., 2019). The central pacemaker is located in the hypothalamic suprachiasmatic nucleus (SCN) and consists of multiple populations of oscillating neurons and astrocytes; these cells are integrated as a single circadian unit and output a coordinated circadian signal (Herzog et al., 2017; Hastings et al., 2018; Brancaccio et al., 2019). SCN forwards the rhythmic signal *via* the hypothalamus-pituitary-adrenal (HPA)-axis and the autonomic nervous system, which align the circadian clocks throughout the organism with the external environmental cues, such as light (Kalsbeek et al., 2012; Koronowski and Sassone-Corsi, 2021). At the molecular level, the core clock machinery exists in almost all cells and is a self-sustaining system based on transcription-translation feedback loops (TTFLs); molecular clocks can generate circadian rhythms autonomously without the need of an external signal (Reppert and Weaver, 2001; Dudek and Meng, 2014). The transcriptional factors brain and muscle ARNT-like 1 (Bmal1)/circadian locomotor output cycles Kaput (Clock) heterodimer activate the expression of genes that containing clock regulatory elements (E-box) in their promoters, including *Period circadian clock* (*Per1/2/3*), *Cryptochrome* (*Cry1/2*), RAR-related orphan receptor alpha (*Ror*α), *Rev-erb*α, and D site albumin promoter binding protein (*Dbp*) genes. Per and Cry, in turn, inhibit the activity of the Bmal1/Clock complex; while the opposing function of nuclear receptors Rorα and Rev-erbα fine-tune the transcription of *Bmal1* and nuclear factor interleukin 3 (*Nfil3*, also known as E4BP4) *via* activation and repression, respectively (Gekakis et al., 1998; Sato et al., 2004; Ripperger and Schibler, 2006; Haque et al., 2019). DBP and NFIL3 synergistically regulate the expression of *Per* (Ohno et al., 2007; Yamajuku et al., 2011; Curtis et al., 2014) (**Figure 1**).

Except for autoregulation, circadian clocks control the rhythmic expression of widespread genes and proteins throughout the body, thus affect numerous physiological processes (Duffield, 2003; Bass and Takahashi, 2010; Gerstner and Yin, 2010; Curtis et al., 2014; Zhang et al., 2014; Rahman et al., 2015; Man et al., 2016; Loizides-Mangold et al., 2017; Stenvers et al., 2019b; Barca-Mayo et al., 2019). For example, synaptic plasticity and density vary according to time-of-day and circadian clock-Bmal1 deficiency in the brain impairs neuronal function and cognitive performance (Perez-Cruz et al., 2009; Musiek et al., 2013; Jasinska et al., 2015; Krzeptowski et al., 2018; Hasegawa et al., 2019). Transcripts of inflammatory cytokines and host immune responses also show a diurnal variation, while disruption of the circadian clock leads to dysfunction of immunity (Halberg et al., 1960; Bellet et al., 2013; Fonken et al., 2015; Deng et al., 2018). Moreover, circadian clock participates in diverse metabolic processes ranging from glucose transport to gluconeogenesis, lipogenesis, and mitochondrial processes (such as morphological changes, mitochondrial biogenesis, respiration, and oxidative phosphorylation) in both peripheral tissues and the brain (Kohsaka, 2007; Bass and Takahashi, 2010; Kawai et al., 2010; Zhang et al., 2010; Schiaffino et al., 2016; Robles et al., 2017; de Goede et al., 2018). Therefore, disruption of the cell-autonomous clock network has a profound influence on immune responses, energy metabolism, and cognition in mammals (Schiaffino et al., 2016; Stenvers et al., 2019b).

To maintain daily oscillations of circadian clocks to approximately 24 h, the negative-feedback loop of clock network requires a crucial delay between activation and repression of transcription, which is achieved by post-translational modifications (PTMs). For example, transcription repressor Per2 is phosphorylated by casein kinase Iε (CKIε) and targeted for ubiquitin-mediated degradation, while Cry1 is phosphorylated and destabilized by adenosine monophosphate-activated protein kinase (AMPK) (Eide et al., 2005; Lamia et al., 2009). Transcription activator Bmal1 is phosphorylated by mitogen-activated protein kinase (MAPK) and glycogen synthase kinase-3β (GSK3β) (Sanada et al., 2002; Sahar et al., 2010); while Clock possesses intrinsic histone acetyltransferase (HAT) activity indicated a close link between clock machinery and chromatin remolding (Doi et al., 2006). The PTMs modulated by phosphorylation, ubiquitylation, sumoylation, acetylation, and epigenetic modification affect the stability and nuclear translocation of core clock proteins, thus control the time-specific transcription of clock genes (Gallego and Virshup, 2007; Bellet and Sassone-Corsi, 2010; Shi et al., 2016). Protein phosphorylation is the widely reported PTM of the core clocks and plays a key role in generation of circadian rhythms (Reischl and Kramer, 2011). It’s also known that epigenetic modification uses cellular metabolites as a source, which indicates the implication of cellular metabolism in PTMs (Borrelli et al., 2008). Notably, rhythmic oscillations of peroxiredoxins, highly conserved antioxidant proteins, have been observed in both mice and cultured human red blood cells (naturally no nucleus or DNA) (O’Neill and Reddy, 2011; Edgar et al., 2012). These studies indicate the essential role of non-transcriptional dependent redox oscillations in generation of circadian rhythms (Rey and Reddy, 2015). Besides, the redox state can regulate circadian clocks and is also relevant for their functions (Rutter et al., 2001; Fleischhacker et al., 2018; Sundar et al., 2018; Uriz-Huarte et al., 2020). Moreover, the expression pattern of clock genes varies in a tissue-specific manner, which may determine the functional difference of some clock components in different tissues (Ko and Takahashi, 2006).

## Effect of Circadian Clock on Peripheral Immune Response

The immune system protects against infections and tissue injury, which has beneficial effects to the host. However, the dysregulated immune response can lead to chronic inflammation, tissue damage, and endotoxin shock (Medzhitov, 2008). While chronic inflammation also presents in tissue stress and malfunction conditions, such as type 2 diabetes, obesity, and neurodegenerative and neuropsychiatric diseases; the persistent low-grade inflammation, in turn, contributes to the further progression of these diseases (DeLegge and Smoke, 2008; Donath and Shoelson, 2011; Cox et al., 2015). Growing evidence shows that circadian clock controls multiple immune functions, such as trafficking of immune cells, pathogen recognition, phagocytic capacity, and secretion of inflammatory cytokines, chemokines, and complement factors (Keller et al., 2009; Curtis et al., 2014; Man et al., 2016; Timmons et al., 2020). Additionally, the mortality caused by lethal bacteria varies according to the time of infection (Halberg et al., 1960; Shackelford and Feigin, 1973; Marpegan et al., 2009). Here, we review the cell-specific disruption of the circadian clock-induced dysfunction of innate and adaptive immunity in peripheral immune cells (Sato et al., 2014; Nakazato et al., 2017; Deng et al., 2018) (**Table 1**).

**Table 1:** Effect of the circadian clock on peripheral innate and adaptive immunity.

Cell type	Target	Stimulation	Phenotype	Mouse/ cell Model	Ref.
**Peritoneal macrophage**	Bmal1	LPS	Increased IL6, CXCL1, TNFα, MCP1/CCL2, and reduced IL10	Myeloid Bmal1^-/-^ mouse	(Curtis et al., 2015)
**BMDM**	Bmal1	LPS	Increased IL6, CXCL1, TNFα, MCP1/CCL2, and *Il1b*	Myeloid Bmal1^-/-^ mouse	(Curtis et al., 2015; Early et al., 2018)
**Alveolar macrophage**	Bmal1	*S. aureus* infection	Increased phagocytosis and reduced pro- and anti-inflammatory cytokines in the lung	Myeloid Bmal1^-/-^ mouse	(Kitchen et al., 2020)
**Peritoneal macrophage**	Bmal1	*S. aureus* or *S. pneumoniae* infection	Increased phagocytosis	Myeloid Bmal1^-/-^ mouse	(Kitchen et al., 2020)
**BMDM**	Clock	LPS	Reduction of *Il-6*, *Il1b*, *Tnf*α, *Ifn-*β, and *Ccl2* and reduced secretion of IL-6 and TNFα	Clock^-/-^ mouse	(Bellet et al., 2013)
**BMDM**	Clock	*Salmonella* infection	Decreased secretion of IL-6 and IL-1β	Clock^-/-^ mouse	(Bellet et al., 2013)
**Peritoneal macrophage**	Per2	TLR9 ligand	Less TNFα and IL12 production	*Per2* mutant mouse	(Silver et al., 2012)
**BMDM**	Cry1 and Cry2	LPS	Increased secretion of TNFα and IL-6	*Cry1−/−;Cry2−/−* mouse	(Narasimamurthy et al., 2012)
**Peritoneal** **macrophage**	Rev-erbα	LPS/ None	Increased *Ccl2* gene expression	*Rev-erbα*^−/−^ mouse	(Sato et al., 2014)
**MDM, alveolar** **macrophage, or THP-1**	Rev-erbα	LPS	Decreased or increased IL-6 level	Rev-erbα agonist or knockdown in human cell	(Gibbs et al., 2012)
**Monocyte**	Bmal1	*Listeria monocytogenes* infection	Increased inflammatory cytokines and monocyte-attracting chemokines in serum	Myeloid Bmal1^-/-^ mouse	(Nguyen et al., 2013)
**Monocyte and macrophage**	Bmal1	Diet-induced obesity and atherosclerosis	Exacerbation or reduction chronic inflammation	Myeloid Bmal1^-/-^ mouse	(Nguyen et al., 2013; Huo et al., 2017; Yang et al., 2020)
**THP-1**	Rorα	LPS/ None	Increased expression of TNFα, IL-1β, and IL-6	Deletion of Rorα in THP-1	(Moharrami et al., 2018)
**NK and DC**	Nfil3	None	Lack of NK and CD8α(+) conventional DCs	Nfil3-/- mouse	(Gascoyne et al., 2009; Kashiwada et al., 2011)
**CD4+ T cell**	Rorα	Inflammatory condition	Reduced IL-17 production	Rorα-/- mouse	(Yang et al., 2008)
**CD4+ T cell**	Rorγt	None	Reduced Th17 cells and IL17 expression	Rorγ−/− mouse	(Ivanov et al., 2006)
**Th17 cell**	REV-ERBα	None	Increased inflammatory response	REV-ERBα−/− mouse	(Amir et al., 2018)
**CD4+ T cell**	REV-ERBα	None	Decreased Th17 cell differentiation	T cell-specific REV-ERBα/β−/− mouse	(Chang et al., 2019)
**CD4+ T cell**	Nfil3	None	Increased Th17 cells	Nfil3−/− mouse	(Yu et al., 2013)
**B cell**	Nfil3	None	Impaired IgE production	Nfil3−/− mouse	(Kashiwada et al., 2010)
**Th1 and Th2 cells**	Nfil3	Antigen stimulation	Altered cytokine production	Nfil3−/− mouse/ cell	(Kashiwada et al., 2011; Motomura et al., 2011)
**T and B cells**	Bmal1	None	Abolished the diurnal oscillation of T and B cell numbers in lymph nodes	CD4^+^/ CD19^+^ cell Bmal1^-/-^ mouse	(Druzd et al., 2017)

BMDM, bone marrow-derived macrophage; MDM, monocyte-derived macrophage; THP-1, myelomonocytic cell line; NK, natural killer; DC, dendritic cell; Ref., reference.

In peritoneal macrophages, the transcript of *Bmal1* exhibits diurnal rhythm with the peak at the dark phase (Deng et al., 2018; Early et al., 2018; Lee et al., 2020). Bmal1 deficient peritoneal macrophages or bone marrow-derived macrophages (BMDMs) show increased production of IL6, CXCL1, TNFα, MCP1/CCL2, or *Il1b* expression upon bacterial endotoxin-lipopolysaccharide (LPS) stimulation; both NRF2 and microRNA miR-155 are implicated in Bmal1 control of inflammatory phenotype (Curtis et al., 2015; Early et al., 2018). Myeloid Bmal1 deficient mice show increased IL-1β levels in the serum after LPS administration and increased mortality response to experimental or LPS-induced sepsis (Curtis et al., 2015; Deng et al., 2018; Early et al., 2018). However, deletion of Bmal1 in macrophages protects mice from pneumococcal infection through increased phagocytosis of macrophages and reduced pro-inflammatory cytokines in the lungs (Kitchen et al., 2020). Additionally, *Clock* deficient BMDMs exhibit reduced expression of proinflammatory genes *Il-6*, *Il1b*, *Tnf*α, *Ifn-*β, and *Ccl2* upon LPS stimulation and decreased secretion of IL-6 and IL-1β after *Salmonella* infection (Bellet et al., 2013). Per2 mutation reduces the Toll-like receptor 9 (TLR9)-dependent TNFα and IL12 levels in peritoneal macrophages upon challenge with TLR9 ligand (Silver et al., 2012). Cry1 and Cry2 deficiency increases the secretion of TNFα and IL-6 in BMDMs upon LPS stimulation by binding to adenylyl cyclase and subsequently regulating cAMP levels and protein kinase A and NF-κB activity (Narasimamurthy et al., 2012). Moreover, Rev-erbα inhibits the expression of Chemokine (C-C motif) ligand 2 (*Ccl2*) in peritoneal macrophages and represses the production of IL-6 in human macrophages response to LPS (Gibbs et al., 2012; Sato et al., 2014).

Besides, both clock genes of blood monocytes and Ly6C^hi^ inflammatory monocytes numbers in blood and spleen show a diurnal variation in mice; depletion of Bma1 in myeloid cells disrupts rhythmic trafficking of Ly6C^hi^ monocytes and induces higher expression of inflammatory cytokines and monocyte-attracting chemokines in serum during *Listeria monocytogenes* infection (Nguyen et al., 2013). *Bmal1* is also involved in monocytes and macrophages-related chronic inflammation in diet-induced obesity and atherosclerosis conditions (Nguyen et al., 2013; Huo et al., 2017; Yang et al., 2020). Additionally, Bmal1 regulates the rhythmic expression of chemokine genes-*Ccl2*, *Ccl8*, and *S100a8* by binding to E-boxes in their promotors in monocytes and peritoneal macrophages (Nguyen et al., 2013). Deletion of Rorα increases the expression of TNFα, IL-1β, and IL-6 in human monocytic cell line (THP-1) (Moharrami et al., 2018). Moreover, NFIL3 is essential for the development of natural killer (NK) cells and CD8α+ conventional dendritic cells (Gascoyne et al., 2009; Kashiwada et al., 2011). It has been reported that approximately 8% of genes are under circadian control in macrophages and more complex molecular mechanisms may be underlying the circadian clock regulation of the innate immune response (Keller et al., 2009).

**Figure 2:**
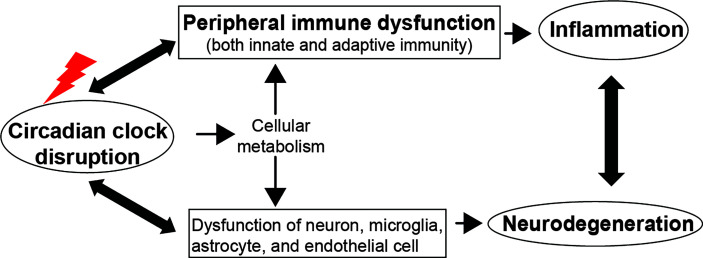
The circadian clock regulates immune function and neurodegeneration. Disruption of the circadian clock machinery leads to immune dysfunction and neurodegeneration, which, in turn, exacerbates the circadian abnormality. The bidirectional communication between systemic inflammation and neurodegeneration deteriorates immune dysfunction and the progression of neurodegenerative diseases. The cellular metabolism pathway could be a mechanism underlying circadian control of immunity and brain function.

Furthermore, circadian clock also modulates adaptive immunity (Downton et al., 2020). Rorα and Rorγt regulate the differentiation of pro-inflammatory T helper 17 (Th17) cells and induce the expression of cytokine-interleukin-17 (IL-17) and IL-17F, whereas Rev-erbα antagonizes the function of Rorγt by binding the same DNA motif (Ivanov et al., 2006; Yang et al., 2008; Korn et al., 2009; Amir et al., 2018; Chang et al., 2019). Transcription repressor NFIL3 also suppresses Th17 cell development by directly binding the *Ror*γ*t* promoter and repressing its activity (Yu et al., 2013). The production of IgE by activated B cells and secretion of cytokines by Th1 and Th2 cells also require the involvement of NFIL3 (Kashiwada et al., 2010; Kashiwada et al., 2011; Motomura et al., 2011; Male et al., 2012). Additionally, lymphocyte migration through lymph nodes and lymph show diurnal variation, which depends on the rhythmic expression of promigratory factors on lymphocytes and can be abolished in lymphocytes loss of Bmal1 (Druzd et al., 2017). However, a study showed that the differentiation of T and B cells and the adaptive immune response are independent of the expression of Bmal1 (Hemmers and Rudensky, 2015). Since the widespread and complex effect of circadian clocks on peripheral immune response, it is necessary to review the role of the molecular clock at the systemic level, especially in circadian disruption and immune dysfunction-related neurodegenerative diseases (**Figure 2**).

## Effect of Circadian Clock on Neurodegeneration

Neurodegeneration is mainly characterized by the progressive loss of structure or function of neurons and finally leads to neuronal death. Increasing evidence suggests multiple cell types in the brain are involved in the progression of neurodegeneration (Heneka et al., 2015; Phatnani and Maniatis, 2015; Liddelow et al., 2017; Hickman et al., 2018; Parodi-Rullan et al., 2019). Microglia provide immune surveillance and shape the neural circuits, while astrocytes regulate neuronal signal transmission. Activated microglia and astrocytes induced-chronic neuroinflammation plays a critical role in the development and progression of neurodegeneration (Hickman et al., 2018; Labzin et al., 2018). Thus, maintenance of brain homeostasis is crucial to avoid and prevent the development of neurodegenerative diseases. Compelling studies indicated that circadian clock modulates brain functions and has a direct effect on the function of brain cells, including neurons, astrocytes, microglia, and endothelial cells (Perez-Cruz et al., 2009; Musiek et al., 2013; Jasinska et al., 2015; Barca-Mayo et al., 2017; Krzeptowski et al., 2018; Hasegawa et al., 2019). Circadian clock disruption is an early symptom of neurodegeneration and also could be a risk factor for the development of neurodegenerative diseases (Hood and Amir, 2017a; Leng et al., 2019). AD and PD are the most common neurodegenerative diseases and best studied in relation to circadian rhythms (Breen et al., 2014; Lauretti et al., 2017; Musiek et al., 2018; Leng et al., 2019). Here, we review the effect of disruption of the circadian clock on the function of brain cells and neurodegeneration.

Circadian clock is associated with microglial immune activity. It has been shown that the circadian clock genes are rhythmically expressed in microglial cells isolated from rodents housed under light/dark conditions (Hayashi et al., 2013; Fonken et al., 2015; Wang et al., 2021). Transcripts of pro-inflammatory cytokines-*Il1b*, *Il6*, and *Tnfa*, also show a time-of-day difference in microglia, with elevated expression during the light phase (Fonken et al., 2015; Wang et al., 2020). Additionally, microglial phagocytosis exhibits time-of-day variation (Choudhury et al., 2020; Griffin et al., 2020). Disruption of the circadian function of microglia may sensitize hippocampal inflammatory response in aged rats (Fonken et al., 2016). Notably, the expression of *Bmal1* is higher during the light phase compared to the dark phase in microglia isolated from mice, which is consistent with transcripts of pro-inflammatory cytokines (Hayashi et al., 2013; Wang et al., 2020). Microglial Bmal1 deficient mice exhibit less *Il6* expression and diminished neuronal damage following middle cerebral artery occlusion than controls (Nakazato et al., 2017). In microglial BV-2 cells, Bmal1 knockdown decreases the expression of pro-inflammatory cytokines (*Il1b*, *Tnfa*, and *Il6*) and increases the expression of anti-inflammatory cytokine-*Il10* upon LPS stimulation (Nakazato et al., 2017; Wang et al., 2020). Moreover, lacking Bmal1 increases the microglial phagocytosis in mice on a high-fat diet as well as during the learning process (Wang et al., 2021). Rev-erbα deficient microglia reveal pro-inflammatory phenotypes and elevated NF-kB activation *via* binding to the promoter regions of related genes; global deletion of Rev-erbα leads to hippocampal microgliosis and neuronal damage in mice (Griffin et al., 2019a). Pharmacologic activation of Rev-erbα inhibits TNFα or LPS-induced pro-inflammatory cytokine expression (Griffin et al., 2019a; Wolff et al., 2020). Furthermore, astrocytes are also involved in innate immunity in the brain and secrete inflammatory mediators. Excessive activation of astrocytes may trigger cell death and neurodegeneration. Rorα deficiency decreases the level of IL-6 at basal conditions in astrocytes, whereas significantly increases IL-6 expression after inflammatory stimulations (Journiac et al., 2009). This study suggests that the circadian clock may exert different effects under different conditions.

In addition, both global *Bmal1* knockout and global Rev-erba knockout increase the expression of complement-related genes in the hippocampus; an enhanced transcript of *C4b* is also observed in the cerebral cortex of neuron and astrocyte *Bmal1* knockout mice (Lananna et al., 2018; Griffin et al., 2019b; Griffin et al., 2020). Elevated expression of *C4b* has also been seen in the neuron-specific *Bmal1* knockout and astrocyte-specific *Bmal1* knockout mice, but not in microglia-specific *Bmal1* knockout mice (Griffin et al., 2020). It’s known that increased *C4b* and *C3* expression is critical for synaptic elimination, which leads to increased microglial phagocytosis and significant synaptic loss in the hippocampal CA3 region of the global Rev-erba mice (Griffin et al., 2020). However, inhibition of Rev-erbα reduces amyloid plaque burden and prevents synaptic loss in 5XFAD mice, an animal model of AD, which may be caused by increased microglial amyloid-β (Aβ) clearance (Lee et al., 2020). Circadian clock also regulates the rhythmic expression of cathepsin S (catS), a microglia-specific lysosomal cysteine protease in the brain, to control the diurnal variation of synaptic strength of the cortical neurons *via* extracellular proteolysis; *Clock* deficiency reduces the gene expression of CatS in microglia cells, while deletion of CatS increases spine density and alters synaptic strength, which leads to hyperlocomotor activity in mice (Hayashi et al., 2013).

Global *Bmal1* knockout mice are complete loss of locomotor circadian rhythm in constant darkness and display impaired hippocampus-dependent learning and memory (Bunger et al., 2000; Wardlaw et al., 2014). These mice also exhibit age-dependent severe astrocyte activation in the cortex and hippocampus, increased expression of pro-inflammatory cytokines-*Tnfa* and cyclooxygenase-2 (COX2, *Ptghs2*), and discrete presynaptic terminal degeneration caused by increased oxidative stress-induced neuronal damage (Musiek et al., 2013; Yang et al., 2016). The astrogliosis and neuropathology are recapitulated in neuron and astrocyte *Bmal1* knockout and *Clock*/neuronal PAS domain protein 2 (*NPAS2*) double-knockout mice, but not in *Per1*/*Per2* double-knockout mice (Musiek et al., 2013). Neuron and astrocyte *Bmal1* knockout mice also exhibit increased total activity in response to novel environment (Musiek et al., 2013). Additionally, global *Bmal1* knockout abolished the rhythmic expression of soluble Aβ peptide in hippocampal interstitial fluid and increases amyloid plaque deposition in a mouse model of β-amyloidosis (*APPPS1-21*), which might be mediated by the increased expression of apolipoprotein E (A*poe*) (Kress et al., 2018). Specific-deletion of *Bmal1* from forebrain excitatory neurons impairs forebrain dependent spatial memory in mice (Snider et al., 2016). Moreover, in MPTP induced-PD mouse model, lacking *Bmal1* exacerbates dopaminergic neuronal loss and glial activation, decreases dopamine transmitter, and aggravates the deficit motor ability (Liu et al., 2020). A study shows that astrocyte-specific *Bmal1* deletion induces cell-autonomous astrocyte activation and inflammatory gene expression, which may be partially mediated by inhibition of glutathione signaling; primary cell co-culture experiment shows that Bmal1 deficient astrocytes promote wild-type neuronal death (Lananna et al., 2018). Deletion of *Bmal1* from astrocytes affects the vasoactive intestinal polypeptide (VIP) expression in SCN and the oscillation of neuronal clocks through GABA signaling; astrocyte-specific *Bmal1* knockout mice display altered circadian locomotor and impaired cognitive phenotypes (Barca-Mayo et al., 2017).

The blood-brain barrier (BBB) isolates the brain from the periphery and is critical for neural function and brain homeostasis. The dysfunction of BBB is implicated in the progression of neurodegeneration in both AD and PD (Kortekaas et al., 2005; Sagare et al., 2013; Parodi-Rullan et al., 2019). The function of BBB is also controlled by circadian clock. Endothelial-specific knockout Bmal1 abolishes the rhythmic efflux by BBB and reduces the efflux from the brain (Zhang et al., 2021). Additionally, *Bmal1* deletion from Nestin-positive cells decreases the pericyte coverage of blood vessels and increases the permeability of BBB (Nakazato et al., 2017). Notably, the brain also contains macrophages in perivascular, meningeal, and choroid plexus, which are distinct from microglia and regulate the function of brain barriers (Mendes-Jorge et al., 2009; Bergen et al., 2015). These macrophages may be also regulated by circadian clock and involved in neurodegeneration (Hawkes and McLaurin, 2009; Baruch et al., 2013; Maleki and Rivest, 2019; Ivan et al., 2020). These studies emphasize the importance of circadian clock in maintaining brain homeostasis. Coordinated response of brain cells promotes the progression of neurodegeneration in response to circadian disruption.

## Effect of Peripheral Immunity on Neurodegeneration

Systemic inflammation or infection has been regarded as a risk factor for neurodegeneration. Past infections may contribute to cognitive impairment and increase the odds of AD and PD development in later life (Dunn et al., 2005; Katan et al., 2013; Semmler et al., 2013; Widmann and Heneka, 2014; Meng et al., 2019). The low dose of peripheral LPS injection induces sickness behaviors and hippocampal inflammation in rats, which are more severe when the administration during the light (i.e. sleep) phase (Fonken et al., 2015). A single LPS injection induced-severe systemic inflammation leads to persistent microglial activation and inflammatory gene expression, as well as long-term cognitive deficits *via* remained elevated NOS2 expression (Weberpals et al., 2009). Besides, peripheral LPS challenge results in the persistent increase of TNFα expression in the brain through TNFα receptors, which leads to microglial proliferation and activation, as well as progressive dopamine neuronal loss (Qin et al., 2007). Importantly, numerous studies revealed that LPS administration is able to recapitulate the pathologies and symptoms of PD in animal models (Liu and Bing, 2011; Zhao et al., 2018). Accumulating evidence suggests that low-grade systemic inflammation caused by tissue stress and malfunction conditions, such as high-fat diet consumption, obesity, and diabetes, also leads to cognitive impairment in both adult humans and rodents (Dunn et al., 2005; Murray et al., 2009; Pistell et al., 2010; Edwards et al., 2011; Holloway et al., 2011; Heyward et al., 2012; Pedditzi et al., 2016). The communication between peripheral inflammatory mediators and the brain has been well studied in animal models. It’s shown that the immunological mediators can activate the local afferent vagus pathway; they can also enter into circulation and directly activate macrophages in circumventricular organs where lack a functional BBB (Tracey, 2002; Perry et al., 2007). Additionally, inflammatory mediators interact with endothelial cells and perivascular macrophages, which may cause dysfunction of BBB and peripheral immune cell infiltration (Laflamme and Rivest, 1999; Bohatschek et al., 2001). The peripheral signals and infiltrated immune cells stimulate microglia and astrocyte activation, which subsequently contributes to neuroinflammation and neuronal damage (Puntener et al., 2012; Widmann and Heneka, 2014).

Moreover, systemic inflammation exacerbates the progression of the ongoing neurodegenerative diseases. A single peripheral LPS injection increases the production of IL-1β and transiently elevates the level of amyloid-β in the brain of Tg2576 and APP/PS1 mice (Sly et al., 2001; Tejera et al., 2019). Besides, LPS treatment triggers microglial activation and peripheral myeloid cell infiltration as well as impairs microglial clearance of amyloid-β in APP/PS1 mice (Tejera et al., 2019). Long-term LPS challenge significantly increases the production of IL-1β and deposition of amyloid precursor protein as well as exacerbates the tau phosphorylation in AD mouse models (Sheng et al., 2003; Kitazawa et al., 2005). Notably, neurodegeneration is a systemic disorder. Both human and animal studies reveal the increased levels of peripheral pro-inflammatory factors and immune cells in the circulation of AD and PD (Collins et al., 2012; Chen et al., 2015; Lai et al., 2017; Yang et al., 2017; King et al., 2018). The blood-borne molecules and peripheral immune cells infiltrate into the brain and exacerbate the progression of neurodegeneration (Zenaro et al., 2015; Kempuraj et al., 2017; St-Amour et al., 2019; Cipollini et al., 2019). In periphery, neutrophil migration from blood to tissue follows a diurnal rhythm, which can be abolished by neutrophil-specific Bmal1 deletion (Adrover et al., 2019). Neutrophil invasion has been seen in the brain of both human and mouse models of AD (5xFAD and 3xTg-AD), while depletion of neutrophils improves memory and reduces AD pathology (Zenaro et al., 2015). Infiltrating T lymphocytes have also been observed in post-mortem brains of patients with frontotemporal dementia and PD (Brochard et al., 2009; Laurent et al., 2017). Depletion of the T cell prevents hippocampal T cell infiltration and reverts spatial memory deficits in a mouse model of AD (THY-Tau22) (Laurent et al., 2017). Th17 cells are also involved in the pathogenesis of AD; neutralization of IL17 cytokine produced by Th17 cells rescues cognitive deficits and ameliorates amyloid-β-induced neuroinflammation in mice (Zhang et al., 2013; Zenaro et al., 2015; Cristiano et al., 2019). Deficiency of mature T lymphocytes or lacking CD4^+^ T cell attenuates dopaminergic neuronal death in the MPTP mouse model of PD (Brochard et al., 2009). Studies also indicated the Th17 cell infiltration in MPTP-treated mice, which exaggerates dopaminergic neuronal loss (Reynolds et al., 2010; Dutta et al., 2019). Moreover, monocytes have been seen in the brain of APP/PS1 mice, but these cells take up Aβ aggregates and transport them back to the bloodstream (Michaud et al., 2013). These infiltrated cell populations have been identified as key players in neurodegenerative diseases. The complex interplay between systemic inflammatory components and dysfunctional brain cells deteriorates the progression of neurodegeneration.

## The Cellular Metabolism - A Mechanism Underlying Circadian Control of Immunity and Neurodegeneration

Recent studies have shown that the metabolic pathway plays a central role in modulation of immunity and brain function (Fukuzumi et al., 1996; Kelly and O’Neill, 2015; Man et al., 2016; O’Neill and Kishton, 2016; O’Neill and Pearce, 2016; Geltink et al., 2018; Wang et al., 2019; Bernier et al., 2020). Immune activity is high energy demand and metabolic disorders are associated with immune dysfunction and neurodegeneration (Bird, 2019; Muddapu et al., 2020). Alteration of cellular metabolisms, such as glycolysis, oxidative phosphorylation, fatty acid, and amino acid metabolism, affects the immune cell response (O’Neill and Kishton, 2016). Enhanced glycolysis and fatty acid synthesis are correlated with peripheral immune cell activation, which leads to a more pro-inflammatory state (Doughty et al., 2006; Posokhova et al., 2008; Rodriguez-Prados et al., 2010; Michalek et al., 2011; Everts et al., 2014; Sharma et al., 2020); whereas, oxidative phosphorylation and fatty acid oxidation have been associated with a more anti-inflammatory phenotype (Michalek et al., 2011; Jha et al., 2015; Patsoukis et al., 2015). Besides, cellular metabolism plays a crucial role in regulating the function of brain cells, which is associated with brain homeostasis. Microglial inflammatory response depends on glycolysis for energy production (Orihuela et al., 2016). Inhibition of glucose transporter 1 reduces glucose uptake and glycolysis in microglia under LPS + IFNγ stimulation, which prevents the upregulation of pro-inflammatory cytokines (Wang et al., 2019). Specific knockdown of lipoprotein lipase (Lpl) in microglia reduces lipid uptake and shifts fuel utilization to glutamine, which attenuates microglial immune response under inflammatory stimuli (Gao et al., 2017). Suppression of glutamine synthetase decreases insulin-mediated glucose uptake and increases the expression of inflammatory mediators in activated microglia (Palmieri et al., 2017). In addition, astrocytes regulate the brain glucose metabolism and provide lactate that is converted from glycogen to supply the high energy requirement of neurons (Suzuki et al., 2011; Weber and Barros, 2015). Astrocytes also rapidly uptake glutamate and metabolize to glutamine to maintain brain homeostasis (Schousboe et al., 2014). Metabolic dysfunction of astrocytes contributes to neurodegeneration (Yan et al., 2013; Oksanen et al., 2019; Sonninen et al., 2020).

Numerous transgenic animal studies revealed that circadian clocks control metabolic processes in extensive tissues (Rudic et al., 2004; Hatanaka et al., 2010; Marcheva et al., 2010; Zhang et al., 2010; Sadacca et al., 2011; Marcheva et al., 2013; Dudek and Meng, 2014; Jha et al., 2015; Schiaffino et al., 2016; Dyar et al., 2018; Sussman et al., 2019). Rhythmic expression of metabolic-related genes optimizes the amount of energy consumption (Wang et al., 2015). It has known that the expression of genes related to microglial glucose and fatty acid uptake shows a time-of-day difference; the expression of glucose transporter member 5 (*Glut5*) and *Lpl* is increased during the dark phase when microglia are more active (Wang et al., 2020). Thus, oscillation in immunity may be driven by rhythmic changes in cellular metabolism. Circadian disruption impacts cellular metabolism and subsequently drives immune dysfunction and neurodegeneration (Carroll et al., 2019; Bernier et al., 2020; Wang et al., 2020). For example, deletion of *Bmal1* in macrophages increases pyruvate kinase M2 expression (PKM2) and lactate production, which is required for expression of the immune checkpoint protein-programmed cell death 1 ligand 1 (PD-L1); mice lacking Bmal1 in myeloid cells are more vulnerable to septic death response to severe infection (Deng et al., 2018). However, knockdown of Bmal1 in microglia decreases expression of *Glu5* and *Lpl*, as well as reduces *Il1b* (Wang et al., 2020). A study also showed that the protein expression of Clock and Bmal1 in astrocytes is elevated in the human cortex with AD; Overexpression of Clock and Bmal1 in human astrocytes significantly inhibits aerobic glycolysis and lactate production but promotes cytotoxicity and functional impairment (Yoo et al., 2020). Thus, maintenance of the homeostasis of the molecular clock network and cellular metabolism is crucial to proper immune function and prevents neurodegeneration.

## Molecule Intervention of Circadian Clock in Inflammation and Neurodegeneration

It’s reported that 82.2% of protein-coding genes identified as druggable targets by the U.S. Food and Drug Administration show rhythmic transcription in at least one human tissue (Mure et al., 2018). Therapeutic targeting circadian core clocks could be a potential strategy to prevent or alleviate inflammation and neurodegenerative diseases (Griffett and Burris, 2013; Ruan et al., 2021) (**Table 2**). Drug molecules that modulate REV-ERBα have been widely studied in different disease models (Uriz-Huarte et al., 2020; Wang et al., 2020). GSK4112-a agonist of REV-ERBα, suppresses IL-6 protein secretion in human MDMs, primary alveolar macrophages, and THP-1 cells in response to LPS; GSK4112 also represses the gene expression of chemokine (*Cxcl11* and *Cxcl6*), and cytokine (*Il19* and *Il10*) in human primary MDMs (Gibbs et al., 2012). Both Hemin-the endogenous activator of REV-ERB and GSK4112 reduce the transcript of *Il6* in THP-1 cells after LPS administration (Raghuram et al., 2007; Gibbs et al., 2012). In contrast, during viral-induced encephalitis, pretreatment of REV-ERBα antagonist-SR8278 significantly reduces the survival rate in mice when infection at the start of the active phase, *via* an increased transcript of pro-inflammatory chemokine-CCL2 (Gagnidze et al., 2016). Surprisingly, REV-ERBα antagonist-GSK1362 also represses the transcript of LPS-induced inflammatory genes-*Il6*, *Ccl2*, and *G-csf* in alveolar macrophages and inhibits the *Il6* gene expression in BMDMs, which may be mediated by stabilizing REV-ERBα protein (Pariollaud et al., 2018). Moreover, inverse agonists of RORα and RORγ, including SR100, ursolic acid, SR2211, SR1555, and ML209, inhibit the development of Th17 cells and reduce associated inflammatory gene expression (Kojetin and Burris, 2014).

**Table 2:** Molecule intervention of circadian clock under different stimulation.

Target	Molecule intervention	Stimulation	Phenotype	Ref.
**REV-ERBα**	Agonist-GSK4112	LPS	Decreased IL-6 in human MDMs, primary alveolar macrophages, and THP-1 cells; Reduced *Cxcl11*, *Cxcl6*, *Il19*, and *Il10* in human MDMs	(Gibbs et al., 2012)
**REV-ERB**	Agonist-GSK4112/ Hemin	LPS	Reduced Il6 in THP-1	(Raghuram et al., 2007; Gibbs et al., 2012)
**REV-ERBα**	Antagonist-SR8278	viral-induced encephalitis	Reduced survival rate in mice; Increased *CCL2* expression	(Gagnidze et al., 2016)
**REV-ERBα**	Antagonist-GSK1362	LPS	Reduced *Il6*, *Ccl2*, and *G-csf* in alveolar macrophages and decreased *Il6* expression in BMDMs	(Pariollaud et al., 2018)
**RORα and RORγ**	Agonists-SR100, ursolic acid, SR2211, SR1555, or ML209	/	Inhibited development of Th17 cells and reduced inflammatory gene expression	(Kojetin and Burris, 2014)
**REV-ERBα**	Agonist-GSK4112/ SR9011	LPS	Inhibited microglial activation and reduced iNOS and COX-2 secretion and *Il6* and *Tnfα* expression	(Nakazato et al., 2017; Guo et al., 2019)
**REV-ERBα**	Agonist-SR9011	/	Reduced phagocytosis, mitochondrial respiration, energy production, and metabolic-related gene expression in primary microglia	(Wolff et al., 2020)
**REV-ERBα**	Agonist-SR9011	TNFα	Decreased *Tnf*α, *Il6*, *Ccl2*, and *Il1*β, and increased Il10 in primary microglia	(Wolff et al., 2020)
**REV-ERBα**	Agonist-SR9009	LPS+ATP	Suppressed IL-1β and Il6 expression in primary microglia	(Griffin et al., 2019a)
**REV-ERBα**	Agonist-SR9009	LPS	Reduced *Il6*, *Tnfa*, and *Il1b* in primary astrocyte	(Griffin et al., 2019a)
**REV-ERBα**	Agonist-SR9009	LPS	Reduced *Il6* and *Ccl2* in hippocampus	(Griffin et al., 2019a)
**REV-ERBα**	Agonist-SR9011, SR9009, and SR10067	/	Increased wakefulness and reduced sleep and anxiety-like behavior in mice	(Banerjee et al., 2014)
**REV-ERBα**	Antagonist-SR8278	/	Induced Mania-like behavior	(Chung et al., 2014)
**REV-ERBα**	Antagonist-SR8278	fAβ1-42	Enhanced microglial phagocytosis and increased fAβ1-42 uptake by BV-2 cells	(Lee et al., 2020)

Besides, REV-ERBα agonists-GSK4112 and SR9011 suppress microglial activation, reduce iNOS and COX-2 secretion, and transcription of *Il6* and *Tnf*α, which may be mediated by repression of nuclear factor kappa B (NF-κB) pathway in LPS-treated microglial cells (Nakazato et al., 2017; Guo et al., 2019). Moreover, SR9011 reduces phagocytosis, mitochondrial respiration, energy production, and metabolic-related gene expression in primary microglial cells; while under TNFα stimulation, SR9011 decreases the transcripts of *Tnf*α, *Il6*, *Ccl2*, and *Il1*β, and increases the expression of *Il10* in primary microglia (Wolff et al., 2020). Activation of Rev-erbα with agonist-SR9009 suppresses IL-1β secretion and *Il6* mRNA expression in primary microglial cells under LPS combined with ATP stimulation, and also significantly reduces the gene expression of *Il6*, *Tnfa*, and *Il1b* in LPS-induced primary astrocytes (Griffin et al., 2019a). SR9009 diminishes LPS-induced transcripts of *Il6* and *Ccl2* in mice hippocampus (Griffin et al., 2019a). REV-ERB agonists (SR9011, SR9009, and SR10067) increase wakefulness and reduce sleep and anxiety-like behavior in mice; notably, the anxiolytic activity of SR10067 is significantly superior than SR9011 (Banerjee et al., 2014; Chung et al., 2014). Whereas, microinfusion of REV-ERBα antagonist-SR8278 into the ventral midbrain induces Mania-like behavior related to the central hyperdopaminergic state (Chung et al., 2014). Additionally, SR8278 promotes microglial phagocytic capacity and increases fAβ1-42 uptake by BV-2 cells (Lee et al., 2020).

## Conclusion

There is clear evidence for the involvement of circadian clock machinery in immune homeostasis and the function of brain cells. Circadian clocks regulate the rhythmic activity of immune cells and govern immune response throughout the body. The function of neuron, astrocyte, and endothelia cells is also regulated by circadian clocks. Circadian disruption leads to immune dysfunction and neurodegeneration. Systemic inflammation is a risk factor and also an accelerator of neurodegenerative diseases, such as AD and PD. Cellular metabolism pathways could be involved in the circadian control of immunity and brain function. Thus, modulation of circadian clocks and cellular metabolism could be a promising therapeutic avenue for inflammatory and neurodegenerative diseases. According to the rhythmic expression of numerous druggable target genes, the time of day for treatment should also be considered to optimize the efficiency. Changing the environment and lifestyle, such as light exposure, exercise, and food intake pattern can entrain the circadian system, which may have a therapeutic effect on inflammation and neurodegeneration (Riemersma-van Der Lek et al., 2008; Figueiro, 2017; Videnovic et al., 2017; Ruan et al., 2021). Moreover, microbial infection, inflammation, and neurodegeneration, in turn, disrupt the clock machinery and subsequently exacerbate the progression of neurodegeneration. Neurodegenerative diseases have a long induction period and no effective therapy. A greater mechanistic understanding of the complex circadian system and the interplay between clock network, inflammation, and neurodegeneration could be critical to identify and manage neurodegenerative diseases in their earliest form. How to pharmacologically target the circadian clock in a cell-type-specific manner from a systemic level needs to be further investigated.

## Author Contributions

X-LW and LL conceived the work and wrote the manuscript. All authors contributed to the article and approved the submitted version.

## Conflict of Interest

The authors declare that the research was conducted in the absence of any commercial or financial relationships that could be construed as a potential conflict of interest.

## Publisher’s Note

All claims expressed in this article are solely those of the authors and do not necessarily represent those of their affiliated organizations, or those of the publisher, the editors and the reviewers. Any product that may be evaluated in this article, or claim that may be made by its manufacturer, is not guaranteed or endorsed by the publisher.

## References

[B1] Adamovich Y. Ladeuix B. Golik M. Koeners M. P. Asher G. (2017). Rhythmic Oxygen Levels Reset Circadian Clocks Through HIF1 Alpha. Cell Metab. 25, 93. doi: 10.1016/j.cmet.2016.09.014 27773695

[B2] Adrover J. M. Del Fresno C. Crainiciuc G. Cuartero M. I. Casanova-Acebes M. Weiss L. A.. (2019). A Neutrophil Timer Coordinates Immune Defense and Vascular Protection. Immunity 50, 390. doi: 10.1016/j.immuni.2019.01.002 30709741

[B3] Amir M. Chaudhari S. Wang R. Campbell S. Mosure S. A. Chopp L. B.. (2018). REV-ERBalpha Regulates TH17 Cell Development and Autoimmunity. Cell Rep. 25, 3733. doi: 10.1016/j.celrep.2018.11.101 30590045 PMC6400287

[B4] Banerjee S. Wang Y. J. Solt L. A. Griffett K. Kazantzis M. Amador A.. (2014). Pharmacological Targeting of the Mammalian Clock Regulates Sleep Architecture and Emotional Behaviour. Nat. Commun. 5, 5759. doi: 10.1038/Ncomms6759 25536025 PMC4495958

[B5] Barca-Mayo O. Boender A. J. Armirotti A. De Pietri Tonelli D. (2019). Deletion of Astrocytic BMAL1 Results in Metabolic Imbalance and Shorter Lifespan in Mice. Glia. 68 (6), 1131–1147 doi: 10.1002/glia.23764 31833591 PMC7496695

[B6] Barca-Mayo O. Pons-Espinal M. Follert P. Armirotti A. Berdondini L. De Pietri Tonelli D. (2017). Astrocyte Deletion of Bmal1 Alters Daily Locomotor Activity and Cognitive Functions *via* GABA Signalling. Nat. Commun. 8, 14336. doi: 10.1038/ncomms14336 28186121 PMC5309809

[B7] Baruch K. Ron-Harel N. Gal H. Deczkowska A. Shifrut E. Ndifon W.. (2013). CNS-Specific Immunity at the Choroid Plexus Shifts Toward Destructive Th2 Inflammation in Brain Aging. Proc. Natl. Acad. Sci. U. S. A. 110, 2264. doi: 10.1073/pnas.1211270110 23335631 PMC3568380

[B8] Bass J. Takahashi J. S. (2010). Circadian Integration of Metabolism and Energetics. Science 330, 1349. doi: 10.1126/science.1195027 21127246 PMC3756146

[B9] Bellet M. M. Deriu E. Liu J. Z. Grimaldi B. Blaschitz C. Zeller M.. (2013). Circadian Clock Regulates the Host Response to Salmonella. Proc. Natl. Acad. Sci. U. S. A. 110, 9897. doi: 10.1073/pnas.1120636110 23716692 PMC3683799

[B10] Bellet M. M. Sassone-Corsi P. (2010). Mammalian Circadian Clock and Metabolism - the Epigenetic Link. J. Cell Sci. 123, 3837. doi: 10.1242/jcs.051649 21048160 PMC2972271

[B11] Bell-Pedersen D. Cassone V. M. Earnest D. J. Golden S. S. Hardin P. E. Thomas T. L.. (2005). Circadian Rhythms From Multiple Oscillators: Lessons From Diverse Organisms. Nat. Rev. Genet. 6, 544. doi: 10.1038/nrg1633 15951747 PMC2735866

[B12] Bergen A. A. Kaing S. ten Brink J. B. Gorgels T. G. Janssen S. F. Bank N. B. (2015). Gene Expression and Functional Annotation of Human Choroid Plexus Epithelium Failure in Alzheimer’s Disease. BMC Genomics 16, 956. doi: 10.1186/S12864-015-2159-Z 26573292 PMC4647590

[B13] Bernier L. P. York E. M. MacVicar B. A. (2020). Immunometabolism in the Brain: How Metabolism Shapes Microglial Function. Trends Neurosci. 43, 854. doi: 10.1016/j.tins.2020.08.008 32958333

[B14] Bird L. (2019). Getting Enough Energy for Immunity. Nat. Rev. Immunol. 19, 269. doi: 10.1038/s41577-019-0159-y 30940933

[B15] Bohatschek M. Werner A. Raivich G. (2001). Systemic LPS Injection Leads to Granulocyte Influx Into Normal and Injured Brain: Effects of ICAM-1 Deficiency. Exp. Neurol. 172, 137. doi: 10.1006/exnr.2001.7764 11681847

[B16] Borrelli E. Nestler E. J. Allis C. D. Sassone-Corsi P. (2008). Decoding the Epigenetic Language of Neuronal Plasticity. Neuron 60, 961. doi: 10.1016/j.neuron.2008.10.012 19109904 PMC2737473

[B17] Brancaccio M. Edwards M. D. Patton A. P. Smyllie N. J. Chesham J. E. Maywood E. S.. (2019). Cell-Autonomous Clock of Astrocytes Drives Circadian Behavior in Mammals. Science 363, 187. doi: 10.1126/science.aat4104 30630934 PMC6440650

[B18] Breen D. P. Vuono R. Nawarathna U. Fisher K. Shneerson J. M. Reddy A. B.. (2014). Sleep and Circadian Rhythm Regulation in Early Parkinson Disease. JAMA Neurol. 71, 589. doi: 10.1001/jamaneurol.2014.65 24687146 PMC4119609

[B19] Brochard V. Combadiere B. Prigent A. Laouar Y. Perrin A. Beray-Berthat V.. (2009). Infiltration of CD4(+) Lymphocytes Into the Brain Contributes to Neurodegeneration in a Mouse Model of Parkinson Disease. J. Clin. Invest. 119, 182. doi: 10.1172/JCI36470 19104149 PMC2613467

[B20] Bunger M. K. Wilsbacher L. D. Moran S. M. Clendenin C. Radcliffe L. A. Hogenesch J. B.. (2000). Mop3 Is an Essential Component of the Master Circadian Pacemaker in Mammals. Cell 103, 1009. doi: 10.1016/S0092-8674(00)00205-1 11163178 PMC3779439

[B21] Camandola S. Mattson M. P. (2017). Brain Metabolism in Health, Aging, and Neurodegeneration. EMBO J. 36, 1474. doi: 10.15252/embj.201695810 28438892 PMC5452017

[B22] Carroll R. G. Timmons G. A. Cervantes-Silva M. P. Kennedy O. D. Curtis A. M. (2019). Immunometabolism Around the Clock. Trends Mol. Med. 25, 612. doi: 10.1016/j.molmed.2019.04.013 31153819

[B23] Cavadini G. Petrzilka S. Kohler P. Jud C. Tobler I. Birchler T.. (2007). TNF-Alpha Suppresses the Expression of Clock Genes by Interfering With E-Box-Mediated Transcription. Proc. Natl. Acad. Sci. U. S. A. 104, 12843. doi: 10.1073/pnas.0701466104 17646651 PMC1937554

[B24] Chang C. Loo C. S. Zhao X. Solt L. A. Liang Y. Q. Bapat S. P.. (2019). The Nuclear Receptor REV-ERB Alpha Modulates Th17 Cell-Mediated Autoimmune Disease. Proc. Natl. Acad. Sci. U. S. A. 116, 18528. doi: 10.1073/pnas.1907563116 31455731 PMC6744854

[B25] Chen Y. H. Qi B. Q. Xu W. F. Ma B. Li L. Chen Q. M.. (2015). Clinical Correlation of Peripheral CD4+-Cell Sub-Sets, Their Imbalance and Parkinson’s Disease. Mol. Med. Rep. 12, 6105. doi: 10.3892/mmr.2015.4136 26239429

[B26] Chitnis T. Weiner H. L. (2017). CNS Inflammation and Neurodegeneration. J. Clin. Invest. 127, 3577. doi: 10.1172/JCI90609 28872464 PMC5617655

[B27] Choudhury M. E. Miyanishi K. Takeda H. Islam A. Matsuoka N. Kubo M.. (2020). Phagocytic Elimination of Synapses by Microglia During Sleep. Glia 68, 44. doi: 10.1002/glia.23698 31429116

[B28] Chung S. Lee E. J. Yun S. Choe H. K. Park S. B. Son H. J.. (2014). Impact of Circadian Nuclear Receptor REV-ERB Alpha on Midbrain Dopamine Production and Mood Regulation. Cell 157, 858. doi: 10.1016/j.cell.2014.03.039 24813609

[B29] Cipollini V. Anrather J. Orzi F. Iadecola C. (2019). Th17 and Cognitive Impairment: Possible Mechanisms of Action. Front. Neuroanat. 13, 95. doi: 10.3389/Fnana.2019.00095 31803028 PMC6877481

[B30] Collins L. M. Toulouse A. Connor T. J. Nolan Y. M. (2012). Contributions of Central and Systemic Inflammation to the Pathophysiology of Parkinson’s Disease. Neuropharmacology 62, 2154. doi: 10.1016/j.neuropharm.2012.01.028 22361232

[B31] Cox A. J. West N. P. Cripps A. W. (2015). Obesity, Inflammation, and the Gut Microbiota. Lancet Diabetes Endo. 3, 207. doi: 10.1016/S2213-8587(14)70134-2 25066177

[B32] Cristiano C. Volpicelli F. Lippiello P. Buono B. Raucci F. Piccolo M.. (2019). Neutralization of IL-17 Rescues Amyloid-Beta-Induced Neuroinflammation and Memory Impairment. Brit. J. Pharmacol. 176, 3544. doi: 10.1111/bph.14586 30673121 PMC6715610

[B33] Crosby P. Hamnett R. Putker M. Hoyle N. P. Reed M. Karam C. J.. (2019). Insulin/IGF-1 Drives PERIOD Synthesis to Entrain Circadian Rhythms With Feeding Time. Cell 177, 896. doi: 10.1016/j.cell.2019.02.017 31030999 PMC6506277

[B34] Curtis A. M. Bellet M. M. Sassone-Corsi P. O’Neill L. A. J. (2014). Circadian Clock Proteins and Immunity. Immunity 40, 178. doi: 10.1016/j.immuni.2014.02.002 24560196

[B35] Curtis A. M. Fagundes C. T. Yang G. R. Palsson-McDermott E. M. Wochal P. McGettrick A. F.. (2015). Circadian Control of Innate Immunity in Macrophages by miR-155 Targeting Bmal1. Proc. Natl. Acad. Sci. U. S. A. 112, 7231. doi: 10.1073/pnas.1501327112 25995365 PMC4466714

[B36] de Goede P. Wefers J. Brombacher E. C. Schrauwen P. Kalsbeek A. (2018). Circadian Rhythms in Mitochondrial Respiration. J. Mol. Endocrinol. 60, R115. doi: 10.1530/Jme-17-0196 29378772 PMC5854864

[B37] DeLegge M. H. Smoke A. (2008). Neurodegeneration and Inflammation. Nutr. Clin. Pract. 23, 35. doi: 10.1177/011542650802300135 18203962

[B38] Deng W. J. Zhu S. Zeng L. Liu J. Kang R. Yang M. H.. (2018). The Circadian Clock Controls Immune Checkpoint Pathway in Sepsis. Cell Rep. 24, 366. doi: 10.1016/j.celrep.2018.06.026 29996098 PMC6094382

[B39] Doi M. Hirayama J. Sassone-Corsi P. (2006). Circadian Regulator CLOCK Is a Histone Acetyltransferase. Cell 125, 497. doi: 10.1016/j.cell.2006.03.033 16678094

[B40] Donath M. Y. Shoelson S. E. (2011). Type 2 Diabetes as an Inflammatory Disease. Nat. Rev. Immunol. 11, 98. doi: 10.1038/nri2925 21233852

[B41] Doughty C. A. Bleiman B. F. Wagner D. J. Dufort F. J. Mataraza J. M. Roberts M. F.. (2006). Antigen Receptor-Mediated Changes in Glucose Metabolism in B Lymphocytes: Role of Phosphatidylinositol 3-Kinase Signaling in the Glycolytic Control of Growth. Blood 107, 4458. doi: 10.1182/blood-2005-12-4788 16449529 PMC1895797

[B42] Downton P. Early J. O. Gibbs J. E. (2020). Circadian Rhythms in Adaptive Immunity. Immunology 161, 268. doi: 10.1111/imm.13167 31837013 PMC7692252

[B43] Druzd D. Matveeva O. Ince L. Harrison U. He W. Y. Schmal C.. (2017). Lymphocyte Circadian Clocks Control Lymph Node Trafficking and Adaptive Immune Responses. Immunity 46, 120. doi: 10.1016/j.immuni.2016.12.011 28087238 PMC5263259

[B44] Dudek M. Meng Q. J. (2014). Running on Time: The Role of Circadian Clocks in the Musculoskeletal System. Biochem. J. 463, 1. doi: 10.1042/BJ20140700 25195734 PMC4157581

[B45] Duffield G. E. (2003). DNA Microarray Analyses of Circadian Timing: The Genomic Basis of Biological Time. J. Neuroendocrinol. 15, 991. doi: 10.1046/j.1365-2826.2003.01082.x 12969245

[B46] Dunn N. Mullee M. Perry V. H. Holmes C. (2005). Association Between Dementia and Infectious Disease: Evidence From a Case-Control Study. Alzheimer Dis. Assoc. Disord. 19, 91. doi: 10.1097/01.wad.0000165511.52746.1f 15942327

[B47] Dutta D. Kundu M. Mondal S. Roy A. Ruehl S. Hall D. A.. (2019). RANTES-Induced Invasion of Th17 Cells Into Substantia Nigra Potentiates Dopaminergic Cell Loss in MPTP Mouse Model of Parkinson’s Disease. Neurobiol. Dis. 132, 104575. doi: 10.1016/J.Nbd.2019.104575 31445159 PMC6834904

[B48] Dyar K. A. Hubert M. J. Mir A. A. Ciciliot S. Lutter D. Greulich F.. (2018). Transcriptional Programming of Lipid and Amino Acid Metabolism by the Skeletal Muscle Circadian Clock. PloS Biol. 16, e2005886. doi: 10.1371/journal.pbio.2005886 30096135 PMC6105032

[B49] Early J. O. Menon D. Wyse C. A. Cervantes-Silva M. P. Zaslona Z. Carroll R. G.. (2018). Circadian Clock Protein BMAL1 Regulates IL-1beta in Macrophages *via* NRF2. Proc. Natl. Acad. Sci. U. S. A. 115, E8460. doi: 10.1073/pnas.1800431115 30127006 PMC6130388

[B50] Edgar R. S. Green E. W. Zhao Y. W. van Ooijen G. Olmedo M. Qin X. M.. (2012). Peroxiredoxins Are Conserved Markers of Circadian Rhythms. Nature 485, 459. doi: 10.1038/nature11088 22622569 PMC3398137

[B51] Edwards L. M. Murray A. J. Holloway C. J. Carter E. E. Kemp G. J. Codreanu I.. (2011). Short-Term Consumption of a High-Fat Diet Impairs Whole-Body Efficiency and Cognitive Function in Sedentary Men. FASEB J. 25, 1088. doi: 10.1096/fj.10-171983 21106937

[B52] Eide E. J. Woolf M. F. Kang H. Woolf P. Hurst W. Camacho F.. (2005). Control of Mammalian Circadian Rhythm by CKI Epsilon-Regulated Proteasome-Mediated PER2 Degradation. Mol. Cell Biol. 25, 2795. doi: 10.1128/Mcb.25.7.2795-2807.2005 15767683 PMC1061645

[B53] Everts B. Amiel E. Huang S. C. C. Smith A. M. Chang C. H. Lam W. Y.. (2014). TLR-Driven Early Glycolytic Reprogramming *via* the Kinases TBK1-IKK Epsilon Supports the Anabolic Demands of Dendritic Cell Activation. Nat. Immunol. 15, 323. doi: 10.1038/ni.2833 24562310 PMC4358322

[B54] Figueiro M. G. (2017). Light, Sleep and Circadian Rhythms in Older Adults With Alzheimer’s Disease and Related Dementias. Neurodegener. Dis. Man. 7, 119. doi: 10.2217/nmt-2016-0060 PMC5836917 28534696

[B55] Fleischhacker A. S. Carter E. L. Ragsdale S. W. (2018). Redox Regulation of Heme Oxygenase-2 and the Transcription Factor, Rev-Erb, Through Heme Regulatory Motifs. Antioxid. Redox Signal. 29, 1841. doi: 10.1089/ars.2017.7368 28990415 PMC6217750

[B56] Fonken L. K. Frank M. G. Kitt M. M. Barrientos R. M. Watkins L. R. Maier S. F. (2015). Microglia Inflammatory Responses Are Controlled by an Intrinsic Circadian Clock. Brain Behav. Immun. 45, 171. doi: 10.1016/j.bbi.2014.11.009 25433170 PMC4386638

[B57] Fonken L. K. Kitt M. M. Gaudet A. D. Barrientos R. M. Watkins L. R. Maier S. F. (2016). Diminished Circadian Rhythms in Hippocampal Microglia may Contribute to Age-Related Neuroinflammatory Sensitization. Neurobiol. Aging 47, 102. doi: 10.1016/j.neurobiolaging.2016.07.019 27568094 PMC5813798

[B58] Fukuzumi M. Shinomiya H. Shimizu Y. Ohishi F. Utsumi S. (1996). Endotoxin-Induced Enhancement of Glucose Influx Into Murine Peritoneal Macrophages *via* GLUT1. Infect. Immun. 64, 108. doi: 10.1128/Iai.64.1.108-112.1996 8557327 PMC173734

[B59] Gabriel B. M. Zierath J. R. (2019). Circadian Rhythms and Exercise - Re-Setting the Clock in Metabolic Disease. Nat. Rev. Endocrinol. 15, 197. doi: 10.1038/s41574-018-0150-x 30655625

[B60] Gagnidze K. Hajdarovic K. H. Moskalenko M. Karatsoreos I. N. McEwen B. S. Bulloch K. (2016). Nuclear Receptor REV-ERB Alpha Mediates Circadian Sensitivity to Mortality in Murine Vesicular Stomatitis Virus-Induced Encephalitis. Proc. Natl. Acad. Sci. U. S. A. 113, 5730. doi: 10.1073/pnas.1520489113 27143721 PMC4878505

[B61] Gallego M. Virshup D. M. (2007). Post-Translational Modifications Regulate the Ticking of the Circadian Clock. Nat. Rev. Mol. Cell Bio. 8, 139. doi: 10.1038/nrm2106 17245414

[B62] Gao Y. Q. Vidal-Itriago A. Kalsbeek M. J. Layritz C. Garcia-Caceres C. Tom R. Z.. (2017). Lipoprotein Lipase Maintains Microglial Innate Immunity in Obesity. Cell Rep. 20, 3034. doi: 10.1016/j.celrep.2017.09.008 28954222

[B63] Gascoyne D. M. Long E. Veiga-Fernandes H. de Boer J. Williams O. Seddon B.. (2009). The Basic Leucine Zipper Transcription Factor E4BP4 Is Essential for Natural Killer Cell Development. Nat. Immunol. 10, 1118. doi: 10.1038/ni.1787 19749763

[B64] Gekakis N. Staknis D. Nguyen H. B. Davis F. C. Wilsbacher L. D. King D. P.. (1998). Role of the CLOCK Protein in the Mammalian Circadian Mechanism. Science 280, 1564. doi: 10.1126/science.280.5369.1564 9616112

[B65] Geltink R. I. K. Kyle R. L. Pearce E. L. (2018). Unraveling the Complex Interplay Between T Cell Metabolism and Function. Annu. Rev. Immunol. 36, 461. doi: 10.1146/annurev-immunol-042617-053019 29677474 PMC6323527

[B66] Gerstner J. R. Yin J. C. P. (2010). Circadian Rhythms and Memory Formation. Nat. Rev. Neurosci. 11, 577. doi: 10.1038/nrn2881 20648063 PMC6544049

[B67] Gibbs J. E. Blaikley J. Beesley S. Matthews L. Simpson K. D. Boyce S. H.. (2012). The Nuclear Receptor REV-ERB Alpha Mediates Circadian Regulation of Innate Immunity Through Selective Regulation of Inflammatory Cytokines. Proc. Natl. Acad. Sci. U. S. A. 109, 582. doi: 10.1073/pnas.1106750109 22184247 PMC3258648

[B68] Gnocchi D. Bruscalupi G. (2017). Circadian Rhythms and Hormonal Homeostasis: Pathophysiological Implications. Biology-Basel 6 (1), 10. doi: 10.3390/Biology6010010 PMC5372003 28165421

[B69] Griffett K. Burris T. P. (2013). The Mammalian Clock and Chronopharmacology. Bioorg. Med. Chem. Lett. 23, 1929. doi: 10.1016/j.bmcl.2013.02.015 23481644 PMC4864859

[B70] Griffin P. Dimitry J. M. Sheehan P. W. Lananna B. V. Guo C. Robinette M. L.. (2019). Circadian Clock Protein Rev-Erba Regulates Neuroinflammation. Proc. Natl. Acad. Sci. U. S. A. 116, 5102. doi: 10.1073/pnas.1812405116 30792350 PMC6421453

[B71] Griffin P. Dimitry J. M. Sheehan P. W. Lananna B. V. Guo C. Robinette M. L.. (2019). Circadian Clock Protein Rev-Erbalpha Regulates Neuroinflammation. Proc. Natl. Acad. Sci. U. S. A. 116, 5102. doi: 10.1073/pnas.1812405116 30792350 PMC6421453

[B72] Griffin P. Sheehan P. W. Dimitry J. M. Guo C. Kanan M. F. Lee J.. (2020). REV-ERBalpha Mediates Complement Expression and Diurnal Regulation of Microglial Synaptic Phagocytosis. Elife 9, e58765. doi: 10.7554/eLife.58765 33258449 PMC7728439

[B73] Guo D. K. Zhu Y. Sun H. Y. Xu X. Y. Zhang S. Hao Z. B.. (2019). Pharmacological Activation of REV-ERBalpha Represses LPS-Induced Microglial Activation Through the NF-kappaB Pathway. Acta Pharmacol. Sin. 40, 26. doi: 10.1038/s41401-018-0064-0 29950615 PMC6318300

[B74] Halberg F. Johnson E. A. Brown B. W. Bittner J. J. (1960). Susceptibility Rhythm to E. Coli Endotoxin and Bioassay. Proc. Soc. Exp. Biol. Med. 103, 142. doi: 10.3181/00379727-103-25439 14398944

[B75] Haque S. N. Booreddy S. R. Welsh D. K. (2019). Effects of BMAL1 Manipulation on the Brain’s Master Circadian Clock and Behavior. Yale J. Biol. Med. 92, 251. 31249486 PMC6585533

[B76] Hasegawa S. Fukushima H. Hosoda H. Serita T. Ishikawa R. Rokukawa T.. (2019). Hippocampal Clock Regulates Memory Retrieval *via* Dopamine and PKA-Induced GluA1 Phosphorylation. Nat. Commun. 10, 5766. doi: 10.1038/s41467-019-13554-y10.1038/s41467-019-13554-y[pii 31852900 PMC6920429

[B77] Hastings M. H. Maywood E. S. Brancaccio M. (2018). Generation of Circadian Rhythms in the Suprachiasmatic Nucleus. Nat. Rev. Neurosci. 19, 453. doi: 10.1038/s41583-018-0026-z 29934559

[B78] Hatanaka F. Matsubara C. Myung J. Yoritaka T. Kamimura N. Tsutsumi S.. (2010). Genome-Wide Profiling of the Core Clock Protein BMAL1 Targets Reveals a Strict Relationship With Metabolism. Mol. Cell Biol. 30, 5636. doi: 10.1128/MCB.00781-10 20937769 PMC3004277

[B79] Hawkes C. A. McLaurin J. (2009). Selective Targeting of Perivascular Macrophages for Clearance of Beta-Amyloid in Cerebral Amyloid Angiopathy. Proc. Natl. Acad. Sci. U. S. A. 106, 1261. doi: 10.1073/pnas.0805453106 19164591 PMC2633563

[B80] Hayashi Y. Koyanagi S. Kusunose N. Okada R. Wu Z. Tozaki-Saitoh H.. (2013). The Intrinsic Microglial Molecular Clock Controls Synaptic Strength *via* the Circadian Expression of Cathepsin S. Sci. Rep. 3, 2744. doi: 10.1038/srep02744 24067868 PMC3783043

[B81] Hemmers S. Rudensky A. Y. (2015). The Cell-Intrinsic Circadian Clock Is Dispensable for Lymphocyte Differentiation and Function. Cell Rep. 11, 1339. doi: 10.1016/j.celrep.2015.04.058 26004187 PMC4464971

[B82] Heneka M. T. Carson M. J. El Khoury J. Landreth G. E. Brosseron F. Feinstein D. L.. (2015). Neuroinflammation in Alzheimer’s Disease. Lancet Neurol. 14, 388. doi: 10.1016/S1474-4422(15)70016-5 25792098 PMC5909703

[B83] Herzog E. D. Hermanstyne T. Smyllie N. J. Hastings M. H. (2017). Regulating the Suprachiasmatic Nucleus (SCN) Circadian Clockwork: Interplay Between Cell-Autonomous and Circuit-Level Mechanisms. Csh Perspect. Biol. 9 (1), a027706. doi: 10.1101/cshperspect.a027706 PMC5204321 28049647

[B84] Heyward F. D. Walton R. G. Carle M. S. Coleman M. A. Garvey W. T. Sweatt J. D. (2012). Adult Mice Maintained on a High-Fat Diet Exhibit Object Location Memory Deficits and Reduced Hippocampal SIRT1 Gene Expression. Neurobiol. Learn. Memory 98, 25. doi: 10.1016/j.nlm.2012.04.005 PMC3389577 22542746

[B85] Hickman S. Izzy S. Sen P. Morsett L. El Khoury J. (2018). Microglia in Neurodegeneration. Nat. Neurosci. 21, 1359. doi: 10.1038/s41593-018-0242-x 30258234 PMC6817969

[B86] Holloway C. J. Cochlin L. E. Emmanuel Y. Murray A. Codreanu I. Edwards L. M.. (2011). A High-Fat Diet Impairs Cardiac High-Energy Phosphate Metabolism and Cognitive Function in Healthy Human Subjects. Am. J. Clin. Nutr. 93, 748. doi: 10.3945/ajcn.110.002758 21270386

[B87] Hood S. Amir S. (2017). Neurodegeneration and the Circadian Clock. Front. Aging Neurosci. 9, 170. doi: 10.3389/Fnagi.2017.00170 28611660 PMC5447688

[B88] Hood S. Amir S. (2017). The Aging Clock: Circadian Rhythms and Later Life. J. Clin. Invest. 127, 437. doi: 10.1172/JCI90328 28145903 PMC5272178

[B89] Huo M. Y. Huang Y. H. Qu D. Zhang H. S. Wong W. T. Chawla A.. (2017). Myeloid Bmal1 Deletion Increases Monocyte Recruitment and Worsens Atherosclerosis. FASEB J. 31, 1097. doi: 10.1096/fj.201601030R 27927724 PMC6191064

[B90] Ivanov I. I. McKenzie B. S. Zhou L. Tadokoro C. E. Lepelley A. Lafaille J. J.. (2006). The Orphan Nuclear Receptor RORgammat Directs the Differentiation Program of Proinflammatory IL-17+ T Helper Cells. Cell 126, 1121. doi: 10.1016/j.cell.2006.07.035 16990136

[B91] Ivan D. C. Walthert S. Berve K. Steudler J. Locatelli G. (2020). Dwellers and Trespassers: Mononuclear Phagocytes at the Borders of the Central Nervous System. Front. Immunol. 11, 609921. doi: 10.3389/fimmu.2020.609921 33746939 PMC7973121

[B92] Jasinska M. Grzegorczyk A. Woznicka O. Jasek E. Kossut M. Barbacka-Surowiak G.. (2015). Circadian Rhythmicity of Synapses in Mouse Somatosensory Cortex. Eur. J. Neurosci. 42, 2585. doi: 10.1111/ejn.13045 26274013

[B93] Jha P. K. Challet E. Kalsbeek A. (2015). Circadian Rhythms in Glucose and Lipid Metabolism in Nocturnal and Diurnal Mammals. Mol. Cell. Endocrinol. 418, 74. doi: 10.1016/j.mce.2015.01.024 25662277

[B94] Jha A. K. Huang S. C. C. Sergushichev A. Lampropoulou V. Ivanova Y. Loginicheva E.. (2015). Network Integration of Parallel Metabolic and Transcriptional Data Reveals Metabolic Modules That Regulate Macrophage Polarization. Immunity 42, 419. doi: 10.1016/j.immuni.2015.02.005 25786174

[B95] Journiac N. Jolly S. Jarvis C. Gautheron V. Rogard M. Trembleau A.. (2009). The Nuclear Receptor ROR Alpha Exerts a Bi-Directional Regulation of IL-6 in Resting and Reactive Astrocytes. Proc. Natl. Acad. Sci. U. S. A. 106, 21365. doi: 10.1073/pnas.0911782106 19955433 PMC2795519

[B96] Kalsbeek A. van der Spek R. Lei J. Endert E. Buijs R. M. Fliers E. (2012). Circadian Rhythms in the Hypothalamo-Pituitary-Adrenal (HPA) Axis. Mol. Cell. Endocrinol. 349, 20. doi: 10.1016/j.mce.2011.06.042 21782883

[B97] Kashiwada M. Cassel S. L. Colgan J. D. Rothman P. B. (2011). NFIL3/E4BP4 Controls Type 2 T Helper Cell Cytokine Expression. EMBO J. 30, 2071. doi: 10.1038/emboj.2011.111emboj2011111[pii 21499227 PMC3098483

[B98] Kashiwada M. Levy D. M. McKeag L. Murray K. Schroder A. J. Canfield S. M.. (2010). IL-4-Induced Transcription Factor NFIL3/E4BP4 Controls IgE Class Switching. Proc. Natl. Acad. Sci. U. S. A. 107, 821. doi: 10.1073/pnas.0909235107 20080759 PMC2818942

[B99] Kashiwada M. Pham N. L. L. Pewe L. L. Harty J. T. Rothman P. B. (2011). NFIL3/E4BP4 Is a Key Transcription Factor for CD8 Alpha(+) Dendritic Cell Development. Blood 117, 6193. doi: 10.1182/blood-2010-07-295873 21474667 PMC3122942

[B100] Katan M. Moon Y. P. Paik M. C. Sacco R. L. Wright C. B. Elkind M. S. (2013). Infectious Burden and Cognitive Function: The Northern Manhattan Study. Neurology 80, 1209. doi: 10.1212/WNL.0b013e3182896e7980/13/1209 23530151 PMC3691781

[B101] Kawai M. Green C. B. Lecka-Czernik B. Douris N. Gilbert M. R. Kojima S.. (2010). A Circadian-Regulated Gene, Nocturnin, Promotes Adipogenesis by Stimulating PPAR-Gamma Nuclear Translocation. Proc. Natl. Acad. Sci. U. S. A. 107, 10508. doi: 10.1073/pnas.1000788107 20498072 PMC2890788

[B102] Keller M. Mazuch J. Abraham U. Eom G. D. Herzog E. D. Volk H. D.. (2009). A Circadian Clock in Macrophages Controls Inflammatory Immune Responses. Proc. Natl. Acad. Sci. U. S. A. 106, 21407. doi: 10.1073/pnas.0906361106 19955445 PMC2795539

[B103] Kelly B. O’Neill L. A. J. (2015). Metabolic Reprogramming in Macrophages and Dendritic Cells in Innate Immunity. Cell Res. 25, 771. doi: 10.1038/cr.2015.68 26045163 PMC4493277

[B104] Kempuraj D. Thangavel R. Selvakumar G. P. Zaheer S. Ahmed M. E. Raikwar S. P.. (2017). Brain and Peripheral Atypical Inflammatory Mediators Potentiate Neuroinflammation and Neurodegeneration. Front. Cell. Neurosci. 11, 216. doi: 10.3389/Fncel.2017.00216 28790893 PMC5522882

[B105] King E. O’Brien J. T. Donaghy P. Morris C. Barnett N. Olsen K.. (2018). Peripheral Inflammation in Prodromal Alzheimer’s and Lewy Body Dementias. J. Neurol. Neurosur. Ps. 89, 339. doi: 10.1136/jnnp-2017-317134 PMC5869446 29248892

[B106] Kitazawa M. Oddo S. Yamasaki T. R. Green K. N. LaFerla F. M. (2005). Lipopolysaccharide-Induced Inflammation Exacerbates Tau Pathology by a Cyclin-Dependent Kinase 5-Mediated Pathway in a Transgenic Model of Alzheimer’s Disease. J. Neurosci. 25, 8843. doi: 10.1523/Jneurosci.2868-05.2005 16192374 PMC6725603

[B107] Kitchen G. B. Cunningham P. S. Poolman T. M. Iqbal M. Maidstone R. Baxter M.. (2020). The Clock Gene Bmal1 Inhibits Macrophage Motility, Phagocytosis, and Impairs Defense Against Pneumonia. Proc. Natl. Acad. Sci. U. S. A. 117 (3), 1543–1551. doi: 10.1073/pnas.1915932117 31900362 PMC6983378

[B108] Kohsaka A. (2007). Bass J. A Sense of Time: How Molecular Clocks Organize Metabolism. Trends Endocrin. Met. 18, 4. doi: 10.1016/j.tem.2006.11.005 17140805

[B109] Kohsaka A. Laposky A. D. Ramsey K. M. Estrada C. Joshu C. Kobayashi Y.. (2007). High-Fat Diet Disrupts Behavioral and Molecular Circadian Rhythms in Mice. Cell Metab. 6, 414. doi: 10.1016/j.cmet.2007.09.006 17983587

[B110] Kojetin D. J. Burris T. P. (2014). REV-ERB and ROR Nuclear Receptors as Drug Targets. Nat. Rev. Drug Discov. 13, 197. doi: 10.1038/nrd4100 24577401 PMC4865262

[B111] Korn T. Bettelli E. Oukka M. Kuchroo V. K. (2009). IL-17 and Th17 Cells. Annu. Rev. Immunol. 27, 485. doi: 10.1146/annurev.immunol.021908.132710 19132915

[B112] Koronowski K. B. Sassone-Corsi P. (2021). Communicating Clocks Shape Circadian Homeostasis. Science 371 (6530), eabd0951. doi: 10.1126/science.abd0951371/6530/eabd0951 33574181 PMC8123919

[B113] Kortekaas R. Leenders K. L. van Oostrom J. C. H. Vaalburg W. Bart J. Willemsen A. T. M.. (2005). Blood-Brain Barrier Dysfunction in Parkinsonian Midbrain *In Vivo* . Ann. Neurol. 57, 176. doi: 10.1002/ana.20369 15668963

[B114] Ko C. H. Takahashi J. S. (2006). Molecular Components of the Mammalian Circadian Clock. Hum. Mol. Genet. 15, R271. doi: 10.1093/hmg/ddl207 16987893

[B115] Kress G. J. Liao F. Dimitry J. Cedeno M. R. FitzGerald G. A. Holtzman D. M.. (2018). Regulation of Amyloid-Beta Dynamics and Pathology by the Circadian Clock. J. Exp. Med. 215, 1059. doi: 10.1084/jem.20172347 29382695 PMC5881473

[B116] Krzeptowski W. Hess G. Pyza E. (2018). Circadian Plasticity in the Brain of Insects and Rodents. Front. Neural Circuit. 12, 32. doi: 10.3389/Fncir.2018.00032 PMC5942159 29770112

[B117] Labzin L. I. Heneka M. T. Latz E. (2018). Innate Immunity and Neurodegeneration. Annu. Rev. Med. 69, 437. doi: 10.1146/annurev-med-050715-104343 29106805

[B118] Laflamme N. Rivest S. (1999). Effects of Systemic Immunogenic Insults and Circulating Proinflammatory Cytokines on the Transcription of the Inhibitory Factor kappaB Alpha Within Specific Cellular Populations of the Rat Brain. J. Neurochem. 73, 309. doi: 10.1046/j.1471-4159.1999.0730309.x 10386984

[B119] Lai K. S. P. Liu C. S. Rau A. Lanctot K. L. Kohler C. A. Pakosh M.. (2017). Peripheral Inflammatory Markers in Alzheimer’s Disease: A Systematic Review and Meta-Analysis of 175 Studies. J. Neurol. Neurosur. Ps. 88, 876. doi: 10.1136/jnnp-2017-316201 28794151

[B120] Lamia K. A. Sachdeva U. M. DiTacchio L. Williams E. C. Alvarez J. G. Egan D. F.. (2009). AMPK Regulates the Circadian Clock by Cryptochrome Phosphorylation and Degradation. Science 326, 437. doi: 10.1126/science.1172156 19833968 PMC2819106

[B121] Lananna B. V. Nadarajah C. J. Izumo M. Cedeno M. R. Xiong D. D. Dimitry J.. (2018). Cell-Autonomous Regulation of Astrocyte Activation by the Circadian Clock Protein Bmal1. Cell Rep. 25, 1. doi: 10.1016/j.celrep.2018.09.015 30282019 PMC6221830

[B122] Laurent C. Dorothee G. Hunot S. Martin E. Monnet Y. Duchamp M.. (2017). Hippocampal T Cell Infiltration Promotes Neuroinflammation and Cognitive Decline in a Mouse Model of Tauopathy. Brain 140, 184. doi: 10.1093/brain/aww270 27818384 PMC5382942

[B123] Lauretti E. Di Meco A. Merali S. Pratico D. (2017). Circadian Rhythm Dysfunction: A Novel Environmental Risk Factor for Parkinson’s Disease. Mol. Psychiatr. 22, 280. doi: 10.1038/mp.2016.47 27046648

[B124] Lee J. Kim D. E. Griffin P. Sheehan P. W. Kim D. H. Musiek E. S.. (2020). Inhibition of REV-ERBs Stimulates Microglial Amyloid-Beta Clearance and Reduces Amyloid Plaque Deposition in the 5XFAD Mouse Model of Alzheimer’s Disease. Aging Cell. 19 (2), e13078. doi: 10.1111/acel.13078 31800167 PMC6996949

[B125] Leng Y. Musiek E. S. Hu K. Cappuccio F. P. Yaffe K. (2019). Association Between Circadian Rhythms and Neurodegenerative Diseases. Lancet Neurol. 18, 307. doi: 10.1016/S1474-4422(18)30461-7 30784558 PMC6426656

[B126] Liddelow S. A. Guttenplan K. A. Larke L. E. C. Bennett F. C. Bohlen C. J. Schirmer L.. (2017). Neurotoxic Reactive Astrocytes Are Induced by Activated Microglia. Nature 541, 481. doi: 10.1038/nature21029 28099414 PMC5404890

[B127] Liu M. Bing G. Y. (2011). Lipopolysaccharide Animal Models for Parkinson’s Disease. Parkinsons Dis.-Us. 2011, 327089. doi: 10.4061/2011/327089 PMC3096023 21603177

[B128] Liu W. W. Wei S. Z. Huang G. D. Liu L. B. Gu C. Shen Y.. (2020). BMAL1 Regulation of Microglia-Mediated Neuroinflammation in MPTP-Induced Parkinson’s Disease Mouse Model. FASEB J. 34, 6570. doi: 10.1096/fj.201901565RR 32246801

[B129] Loizides-Mangold U. Perrin L. Vandereycken B. Betts J. A. Walhin J. P. Templeman I.. (2017). Lipidomics Reveals Diurnal Lipid Oscillations in Human Skeletal Muscle Persisting in Cellular Myotubes Cultured *In Vitro* . Proc. Natl. Acad. Sci. U. S. A. 114, E8565. doi: 10.1073/pnas.1705821114 28973848 PMC5642690

[B130] Maleki A. F. Rivest S. (2019). Innate Immune Cells: Monocytes, Monocyte-Derived Macrophages and Microglia as Therapeutic Targets for Alzheimer’s Disease and Multiple Sclerosis. Front. Cell. Neurosci. 13, 355. doi: 10.3389/Fncel.2019.00355 31427930 PMC6690269

[B131] Male V. Nisoli I. Gascoyne D. M. Brady H. J. M. (2012). E4BP4: An Unexpected Player in the Immune Response. Trends Immunol. 33, 98. doi: 10.1016/j.it.2011.10.002 22075207

[B132] Man K. Loudon A. Chawla A. (2016). Immunity Around the Clock. Science 354, 999. doi: 10.1126/science.aah4966 27885005 PMC5247264

[B133] Manoogian E. N. C. Panda S. (2017). Circadian Rhythms, Time-Restricted Feeding, and Healthy Aging. Ageing Res. Rev. 39, 59. doi: 10.1016/j.arr.2016.12.006 28017879 PMC5814245

[B134] Marcheva B. Ramsey K. M. Buhr E. D. Kobayashi Y. Su H. Ko C. H.. (2010). Disruption of the Clock Components CLOCK and BMAL1 Leads to Hypoinsulinaemia and Diabetes. Nature 466, 627. doi: 10.1038/nature09253 20562852 PMC2920067

[B135] Marcheva B. Ramsey K. M. Peek C. B. Affinati A. Maury E. Bass J. (2013). Circadian Clocks and Metabolism. Handb. Exp. Pharmacol. 127–155. doi: 10.1007/978-3-642-25950-0_6 PMC4089089 23604478

[B136] Marpegan L. Leone M. J. Katz M. E. Sobrero P. M. Bekinstein T. A. Golombek D. A. (2009). Diurnal Variation in Endotoxin-Induced Mortality in Mice: Correlation With Proinflammatory Factors. Chronobiol. Int. 26, 1430. doi: 10.3109/07420520903408358 19916840

[B137] Medzhitov R. (2008). Origin and Physiological Roles of Inflammation. Nature 454, 428. doi: 10.1038/nature07201 18650913

[B138] Mendes-Jorge L. Ramos D. Luppo M. Llombart C. Alexandre-Pires G. Nacher V.. (2009). Scavenger Function of Resident Autofluorescent Perivascular Macrophages and Their Contribution to the Maintenance of the Blood-Retinal Barrier. Invest. Ophth. Vis. Sci. 50, 5997. doi: 10.1167/iovs.09-3515 19608545

[B139] Meng L. Shen L. Ji H. F. (2019). Impact of Infection on Risk of Parkinson’s Disease: A Quantitative Assessment of Case-Control and Cohort Studies. J. Neurovirol. 25, 221. doi: 10.1007/s13365-018-0707-4 30632012

[B140] Michalek R. D. Gerriets V. A. Jacobs S. R. Macintyre A. N. MacIver N. J. Mason E. F.. (2011). Cutting Edge: Distinct Glycolytic and Lipid Oxidative Metabolic Programs Are Essential for Effector and Regulatory CD4(+) T Cell Subsets. J. Immunol. 186, 3299. doi: 10.4049/jimmunol.1003613 21317389 PMC3198034

[B141] Michaud J. P. Bellavance M. A. Prefontaine P. Rivest S. (2013). Real-Time In Vivo Imaging Reveals the Ability of Monocytes to Clear Vascular Amyloid Beta. Cell Rep. 5, 646. doi: 10.1016/j.celrep.2013.10.010 24210819

[B142] Moharrami N. N. Tande E. B. Ryan L. Espevik T. Boyartchuk V. (2018). ROR Alpha Controls Inflammatory State of Human Macrophages. PloS One 13. doi: 10.1371/journal.pone.0207374 PMC6261595 30485323

[B143] Mohawk J. A. Green C. B. Takahashi J. S. (2012). Central and Peripheral Circadian Clocks in Mammals. Annu. Rev. Neurosci. 35, 445. doi: 10.1146/annurev-neuro-060909-153128 22483041 PMC3710582

[B144] Motomura Y. Kitamura H. Hijikata A. Matsunaga Y. Matsumoto K. Inoue H.. (2011). The Transcription Factor E4BP4 Regulates the Production of IL-10 and IL-13 in CD4+ T Cells. Nat. Immunol. 12, 450. doi: 10.1038/ni.2020ni.2020[pii 21460847 PMC3494493

[B145] Muddapu V. R. Dharshini S. A. P. Chakravarthy V. S. Gromiha M. M. (2020). Neurodegenerative Diseases - Is Metabolic Deficiency the Root Cause? Front. Neurosci.-Switz. 14, 213. doi: 10.3389/Fnins.2020.00213 PMC7137637 32296300

[B146] Mure L. S. Le H. D. Benegiamo G. Chang M. W. Rios L. Jillani N.. (2018). Diurnal Transcriptome Atlas of a Primate Across Major Neural and Peripheral Tissues. Science 359, 1232. doi: 10.1126/science.aao0318 PMC5924732 29439024

[B147] Murray A. J. Knight N. S. Cochlin L. E. McAleese S. Deacon R. M. J. Rawlins J. N. P.. (2009). Deterioration of Physical Performance and Cognitive Function in Rats With Short-Term High-Fat Feeding. FASEB J. 23, 4353. doi: 10.1096/fj.09-139691 19667117

[B148] Musiek E. S. Bhimasani M. Zangrilli M. A. Morris J. C. Holtzman D. M. Ju Y. E. (2018). Circadian Rest-Activity Pattern Changes in Aging and Preclinical Alzheimer Disease. JAMA Neurol. 75, 582. doi: 10.1001/jamaneurol.2017.4719 29379963 PMC5885197

[B149] Musiek E. S. Lim M. M. Yang G. R. Bauer A. Q. Oi L. Lee Y.. (2013). Circadian Clock Proteins Regulate Neuronal Redox Homeostasis and Neurodegeneration. J. Clin. Invest. 123, 5389. doi: 10.1172/JCI70317 24270424 PMC3859381

[B150] Nakazato R. Hotta S. Yamada D. Kou M. Nakamura S. Takahata Y.. (2017). The Intrinsic Microglial Clock System Regulates Interleukin-6 Expression. Glia 65, 198. doi: 10.1002/glia.23087 27726182

[B151] Nakazato R. Kawabe K. Yamada D. Ikeno S. Mieda M. Shimba S.. (2017). Disruption of Bmal1 Impairs Blood-Brain Barrier Integrity *via* Pericyte Dysfunction. J. Neurosci. 37, 10052. doi: 10.1523/Jneurosci.3639-16.2017 28912161 PMC6596539

[B152] Narasimamurthy R. Hatori M. Nayak S. K. Liu F. Panda S. Verma I. M. (2012). Circadian Clock Protein Cryptochrome Regulates the Expression of Proinflammatory Cytokines. Proc. Natl. Acad. Sci. U. S. A. 109, 12662. doi: 10.1073/pnas.1209965109 22778400 PMC3411996

[B153] Nguyen K. D. Fentress S. J. Qiu Y. F. Yun K. R. Cox J. S. Chawla A. (2013). Circadian Gene Bmal1 Regulates Diurnal Oscillations of Ly6C(hi) Inflammatory Monocytes. Science 341, 1483. doi: 10.1126/science.1240636 23970558 PMC3836670

[B154] Ohno T. Onishi Y. Ishida N. (2007). A Novel E4BP4 Element Drives Circadian Expression of Mperiod2. Nucleic Acids Res. 35, 648. doi: 10.1093/nar/gkl868 17182630 PMC1802629

[B155] Oksanen M. Lehtonen S. Jaronen M. Goldsteins G. Hamalainen R. H. Koistinaho J. (2019). Astrocyte Alterations in Neurodegenerative Pathologies and Their Modeling in Human Induced Pluripotent Stem Cell Platforms. Cell Mol. Life Sci. 76, 2739. doi: 10.1007/s00018-019-03111-7 31016348 PMC6588647

[B156] O’Neill L. A. J. Kishton R. J. (2016). Rathmell J. A Guide to Immunometabolism for Immunologists. Nat. Rev. Immunol. 16, 553. doi: 10.1038/nri.2016.70 27396447 PMC5001910

[B157] O’Neill L. A. J. Pearce E. J. (2016). Immunometabolism Governs Dendritic Cell and Macrophage Function. J. Exp. Med. 213, 15. doi: 10.1084/jem.20151570 26694970 PMC4710204

[B158] O’Neill J. S. Reddy A. B. (2011). Circadian Clocks in Human Red Blood Cells. Nature 469, 498. doi: 10.1038/nature09702 21270888 PMC3040566

[B159] Orihuela R. McPherson C. A. Harry G. J. (2016). Microglial M1/M2 Polarization and Metabolic States. Brit. J. Pharmacol. 173, 649. doi: 10.1111/bph.13139 25800044 PMC4742299

[B160] Palmieri E. M. Menga A. Lebrun A. Hooper D. C. Butterfield D. A. Mazzone M.. (2017). Blockade of Glutamine Synthetase Enhances Inflammatory Response in Microglial Cells. Antioxid. Redox Sign. 26, 351. doi: 10.1089/ars.2016.6715 PMC5346956 27758118

[B161] Pariollaud M. Gibbs J. E. Hopwood T. W. Brown S. Begley N. Vonslow R.. (2018). Circadian Clock Component REV-ERB Alpha Controls Homeostatic Regulation of Pulmonary Inflammation. J. Clin. Invest. 128, 2281. doi: 10.1172/JCI93910 29533925 PMC5983347

[B162] Parodi-Rullan R. Sone J. Y. Fossati S. (2019). Endothelial Mitochondrial Dysfunction in Cerebral Amyloid Angiopathy and Alzheimer’s Disease. J. Alzheimers Dis. 72, 1019. doi: 10.3233/Jad-190357 31306129 PMC6917858

[B163] Patsoukis N. Bardhan K. Chatterjee P. Sari D. Liu B. L. Bell L. N.. (2015). PD-1 Alters T-Cell Metabolic Reprogramming by Inhibiting Glycolysis and Promoting Lipolysis and Fatty Acid Oxidation. Nat. Commun. 6, 6692. doi: 10.1038/Ncomms7692 25809635 PMC4389235

[B164] Pedditzi E. Peters R. Beckett N. (2016). The Risk of Overweight/Obesity in Mid-Life and Late Life for the Development of Dementia: A Systematic Review and Meta-Analysis of Longitudinal Studies. Age Ageing 45, 737. doi: 10.1093/ageing/afw095 26764391

[B165] Perez-Cruz C. Simon M. Flugge G. Fuchs E. Czeh B. (2009). Diurnal Rhythm and Stress Regulate Dendritic Architecture and Spine Density of Pyramidal Neurons in the Rat Infralimbic Cortex. Behav. Brain Res. 205, 406. doi: 10.1016/j.bbr.2009.07.021 19643147

[B166] Perry V. H. Cunningham C. Holmes C. (2007). Systemic Infections and Inflammation Affect Chronic Neurodegeneration. Nat. Rev. Immunol. 7, 161. doi: 10.1038/nri2015 17220915

[B167] Phatnani H. Maniatis T. (2015). Astrocytes in Neurodegenerative Disease. Csh Perspect. Biol. 7 (11), e0207374. doi: 10.1101/cshperspect.a020628 PMC4448607 25877220

[B168] Pistell P. J. Morrison C. D. Gupta S. Knight A. G. Keller J. N. Ingram D. K.. (2010). Cognitive Impairment Following High Fat Diet Consumption Is Associated With Brain Inflammation. J. Neuroimmunol. 219, 25. doi: 10.1016/j.jneuroim.2009.11.010 20004026 PMC2823983

[B169] Pivovarova O. Jurchott K. Rudovich N. Hornemann S. Ye L. Mockel S.. (2015). Changes of Dietary Fat and Carbohydrate Content Alter Central and Peripheral Clock in Humans. J. Clin. Endocr. Metab. 100, 2291. doi: 10.1210/jc.2014-3868 25822100

[B170] Posokhova E. N. Khoshchenko O. M. Chasovskikh M. I. Pivovarova E. N. Dushkin M. I. (2008). Lipid Synthesis in Macrophages During Inflammation *In Vivo*: Effect of Agonists of Peroxisome Proliferator Activated Receptors Alpha and Gamma and of Retinoid X Receptors. Biochemistry-Moscow+ 73, 296. doi: 10.1134/S0006297908030097 18393765

[B171] Puntener U. Booth S. G. Perry V. H. Teeling J. L. (2012). Long-Term Impact of Systemic Bacterial Infection on the Cerebral Vasculature and Microglia. J. Neuroinflamm. 9, 146. doi: 10.1186/1742-2094-9-146 PMC3439352 22738332

[B172] Qin L. Y. Wu X. F. Block M. L. Liu Y. X. Breese G. R. Hong J. S.. (2007). Systemic LPS Causes Chronic Neuroinflammation and Progressive Neurodegeneration. Glia 55, 453. doi: 10.1002/glia.20467 17203472 PMC2871685

[B173] Raghuram S. Stayrook K. R. Huang P. X. Rogers P. M. Nosie A. K. McClure D. B.. (2007). Identification of Heme as the Ligand for the Orphan Nuclear Receptors REV-ERB Alpha and REV-ERB Beta. Nat. Struct. Mol. Biol. 14, 1207. doi: 10.1038/nsmb1344 18037887 PMC2743565

[B174] Rahman S. A. Castanon-Cervantes O. Scheer F. A. Shea S. A. Czeisler C. A. Davidson A. J.. (2015). Endogenous Circadian Regulation of Pro-Inflammatory Cytokines and Chemokines in the Presence of Bacterial Lipopolysaccharide in Humans. Brain Behav. Immun. 47, 4. doi: 10.1016/j.bbi.2014.11.003 25452149 PMC4430446

[B175] Reischl S. Kramer A. (2011). Kinases and Phosphatases in the Mammalian Circadian Clock. FEBS Lett. 585, 1393. doi: 10.1016/j.febslet.2011.02.038 21376720

[B176] Reppert S. M. Weaver D. R. (2001). Molecular Analysis of Mammalian Circadian Rhythms. Annu. Rev. Physiol. 63, 647. doi: 10.1146/annurev.physiol.63.1.647 11181971

[B177] Reppert S. M. Weaver D. R. (2002). Coordination of Circadian Timing in Mammals. Nature 418, 935. doi: 10.1038/nature00965 12198538

[B178] Reynolds A. D. Stone D. K. Hutter J. A. L. Benner E. J. Mosley R. L. Gendelman H. E. (2010). Regulatory T Cells Attenuate Th17 Cell-Mediated Nigrostriatal Dopaminergic Neurodegeneration in a Model of Parkinson’s Disease. J. Immunol. 184, 2261. doi: 10.4049/jimmunol.0901852 20118279 PMC2824790

[B179] Rey G. Reddy A. B. (2015). Interplay Between Cellular Redox Oscillations and Circadian Clocks. Diabetes Obes. Metab. 17, 55. doi: 10.1111/dom.12519 26332969

[B180] Riemersma-van Der Lek R. F. Swaab D. F. Twisk J. Hol E. M. Hoogendijk W. J. G. Van Someren E. J. W. (2008). Effect of Bright Light and Melatonin on Cognitive and Noncognitive Function in Elderly Residents of Group Care Facilities - A Randomized Controlled Trial. Jama-J. Am. Med. Assoc. 299, 2642. doi: 10.1001/jama.299.22.2642 18544724

[B181] Ripperger J. R. A. Schibler U. (2006). Rhythmic CLOCK-BMAL1 Binding to Multiple E-Box Motifs Drives Circadian Dbp Transcription and Chromatin Transitions. Nat. Genet. 38, 369. doi: 10.1038/ng1738 16474407

[B182] Robles M. S. Humphrey S. J. Mann M. (2017). Phosphorylation Is a Central Mechanism for Circadian Control of Metabolism and Physiology. Cell Metab. 25, 118. doi: 10.1016/j.cmet.2016.10.004 27818261

[B183] Rodriguez-Prados J. C. Traves P. G. Cuenca J. Rico D. Aragones J. Martin-Sanz P.. (2010). Substrate Fate in Activated Macrophages: A Comparison Between Innate, Classic, and Alternative Activation. J. Immunol. 185, 605. doi: 10.4049/jimmunol.0901698 20498354

[B184] Ruan W. Yuan X. Y. Eltzschig H. K. (2021). Circadian Rhythm as a Therapeutic Target. Nat. Rev. Drug Discov. 20 (4), 287–307. doi: 10.1038/s41573-020-00109-w 33589815 PMC8525418

[B185] Rudic R. D. McNamara P. Curtis A. M. Boston R. C. Panda S. Hogenesch J. B.. (2004). BMAL1 and CLOCK, Two Essential Components of the Circadian Clock, Are Involved in Glucose Homeostasis. PloS Biol. 2, e377. doi: 10.1371/journal.pbio.0020377 15523558 PMC524471

[B186] Rutter J. Reick M. Wu L. C. McKnight S. L. (2001). Regulation of Clock and NPAS2 DNA Binding by the Redox State of NAD Cofactors. Science 293, 510. doi: 10.1126/science.10606981060698[pii 11441146

[B187] Sadacca L. A. Lamia K. A. deLemos A. S. Blum B. Weitz C. J. (2011). An Intrinsic Circadian Clock of the Pancreas Is Required for Normal Insulin Release and Glucose Homeostasis in Mice. Diabetologia 54, 120. doi: 10.1007/s00125-010-1920-8 20890745 PMC2995870

[B188] Sagare A. P. Bell R. D. Zhao Z. Ma Q. Y. Winkler E. A. Ramanathan A.. (2013). Pericyte Loss Influences Alzheimer-Like Neurodegeneration in Mice. Nat. Commun. 4, 2932. doi: 10.1038/Ncomms3932 24336108 PMC3945879

[B189] Sahar S. Zocchi L. Kinoshita C. Borrelli E. Sassone-Corsi P. (2010). Regulation of BMAL1 Protein Stability and Circadian Function by GSK3 Beta-Mediated Phosphorylation. PloS One 5 (1), e8561. doi: 10.1371/journal.pone.0008561 20049328 PMC2797305

[B190] Saini R. Jaskolski M. Davis S. J. (2019). Circadian Oscillator Proteins Across the Kingdoms of Life: Structural Aspects. BMC Biol. 17 (1), 13. doi: 10.1186/s12915-018-0623-3 30777051 PMC6378743

[B191] Sanada K. Okano T. Fukada Y. (2002). Mitogen-Activated Protein Kinase Phosphorylates and Negatively Regulates Basic Helix-Loop-Helix-PAS Transcription Factor BMAL1. J. Biol. Chem. 277, 267. doi: 10.1074/jbc.M107850200 11687575

[B192] Sato T. K. Panda S. Miraglia L. J. Reyes T. M. Rudic R. D. McNamara P.. (2004). A Functional Genomics Strategy Reveals Rora as a Component of the Mammalian Circadian Clock. Neuron 43, 527. doi: 10.1016/j.neuron.2004.07.018 15312651

[B193] Sato S. Sakurai T. Ogasawara J. Takahashi M. Izawa T. Imaizumi K.. (2014). A Circadian Clock Gene, Rev-Erbalpha, Modulates the Inflammatory Function of Macrophages Through the Negative Regulation of Ccl2 Expression. J. Immunol. 192, 407. doi: 10.4049/jimmunol.1301982jimmunol.1301982[pii 24307731

[B194] Scheiermann C. Kunisaki Y. Frenette P. S. (2013). Circadian Control of the Immune System. Nat. Rev. Immunol. 13, 190. doi: 10.1038/nri3386 23391992 PMC4090048

[B195] Schiaffino S. Blaauw B. Dyar K. A. (2016). The Functional Significance of the Skeletal Muscle Clock: Lessons From Bmal1 Knockout Models. Skelet. Muscle 6, 33. doi: 10.1186/s13395-016-0107-5107 27752300 PMC5062818

[B196] Schousboe A. Scafidi S. Bak L. K. Waagepetersen H. S. McKenna M. C. (2014). Glutamate Metabolism in the Brain Focusing on Astrocytes. Adv. Neurobiol. 11, 13. doi: 10.1007/978-3-319-08894-5_2 25236722 PMC4667713

[B197] Semmler A. Widmann C. N. Okulla T. Urbach H. Kaiser M. Widman G.. (2013). Persistent Cognitive Impairment, Hippocampal Atrophy and EEG Changes in Sepsis Survivors. J. Neurol. Neurosur. Ps. 84, 62. doi: 10.1136/jnnp-2012-302883 23134661

[B198] Shackelford P. G. Feigin R. D. (1973). Periodicity of Susceptibility to Pneumococcal Infection: Influence of Light and Adrenocortical Secretions. Science 182:285. doi: 10.1126/science.182.4109.285 4147530

[B199] Sharma M. Boytard L. Hadi T. Koelwyn G. Simon R. Ouimet M.. (2020). Enhanced Glycolysis and HIF-1 Alpha Activation in Adipose Tissue Macrophages Sustains Local and Systemic Interleukin-1 Beta Production in Obesity. Sci. Rep. 10, 5555. doi: 10.1038/S41598-020-62272-9 32221369 PMC7101445

[B200] Sheng J. G. Bora S. H. Xu G. Borchelt D. R. Price D. L. Koliatsos V. E. (2003). Lipopolysaccharide-Induced-Neuroinflammation Increases Intracellular Accumulation of Amyloid Precursor Protein and Amyloid Beta Peptide in APPswe Transgenic Mice. Neurobiol. Dis. 14, 133. doi: 10.1016/s0969-9961(03)00069-x 13678674

[B201] Shi G. S. Xie P. C. Qu Z. P. Zhang Z. H. Dong Z. An Y.. (2016). Distinct Roles of HDAC3 in the Core Circadian Negative Feedback Loop Are Critical for Clock Function. Cell Rep. 14, 823. doi: 10.1016/j.celrep.2015.12.076 26776516

[B202] Silver A. C. Arjona A. Walker W. E. Fikrig E. (2012). The Circadian Clock Controls Toll-Like Receptor 9-Mediated Innate and Adaptive Immunity. Immunity 36, 251. doi: 10.1016/j.immuni.2011.12.017 22342842 PMC3315694

[B203] Sly L. M. Krzesicki R. F. Brashler J. R. Buhl A. E. McKinley D. D. Carter D. B.. (2001). Endogenous Brain Cytokine mRNA and Inflammatory Responses to Lipopolysaccharide Are Elevated in the Tg2576 Transgenic Mouse Model of Alzheimer’s Disease. Brain Res. Bull. 56, 581. doi: 10.1016/S0361-9230(01)00730-4 11786245

[B204] Snider K. H. Dziema H. Aten S. Loeser J. Norona F. E. Hoyt K.. (2016). Modulation of Learning and Memory by the Targeted Deletion of the Circadian Clock Gene Bmal1 in Forebrain Circuits. Behav. Brain Res. 308, 222. doi: 10.1016/j.bbr.2016.04.027 27091299 PMC5344043

[B205] Sonninen T. M. Hamalainen R. H. Koskuvi M. Oksanen M. Shakirzyanova A. Wojciechowski S.. (2020). Metabolic Alterations in Parkinson’s Disease Astrocytes. Sci. Rep. 10, 14474. doi: 10.1038/S41598-020-71329-8 32879386 PMC7468111

[B206] St-Amour I. Bosoi C. R. Pare I. Doss P. M. I. A. Rangachari M. Hebert S. S.. (2019). Peripheral Adaptive Immunity of the Triple Transgenic Mouse Model of Alzheimer’s Disease. J. Neuroinflamm. 16 (1), 3. doi: 10.1186/S12974-018-1380-5 PMC6320637 30611289

[B207] Stenvers D. J. Scheer F. A. J. L. Schrauwen P. la Fleur S. E. Kalsbeek A. (2019). Circadian Clocks and Insulin Resistance. Nat. Rev. Endocrinol. 15, 75. doi: 10.1038/s41574-018-0122-1 30531917

[B208] Sundar I. K. Sellix M. T. Rahman I. (2018). Redox Regulation of Circadian Molecular Clock in Chronic Airway Diseases. Free Radic. Biol. Med. 119, 121. doi: 10.1016/j.freeradbiomed.2017.10.383 29097215 PMC5910271

[B209] Sussman W. Stevenson M. Mowdawalla C. Mota S. Ragolia L. Pan X. Y. (2019). BMAL1 Controls Glucose Uptake Through Paired-Homeodomain Transcription Factor 4 in Differentiated Caco-2 Cells. Am. J. Physiol.-Cell Ph. 317, C492. doi: 10.1152/ajpcell.00058.2019 PMC6766619 31216190

[B210] Suzuki A. Stern S. A. Bozdagi O. Huntley G. W. Walker R. H. Magistretti P. J.. (2011). Astrocyte-Neuron Lactate Transport Is Required for Long-Term Memory Formation. Cell 144, 810. doi: 10.1016/j.cell.2011.02.018 21376239 PMC3073831

[B211] Tejera D. Mercan D. Sanchez-Caro J. M. Hanan M. Greenberg D. Soreq H.. (2019). Systemic Inflammation Impairs Microglial A Beta Clearance Through NLRP3 Inflammasome. EMBO J. 38, e101064. doi: 10.15252/embj.2018101064 31359456 PMC6717897

[B212] Timmons G. A. O’Siorain J. R. Kennedy O. D. Curtis A. M. Early J. O. (2020). Innate Rhythms: Clocks at the Center of Monocyte and Macrophage Function. Front. Immunol. 11, 1743. doi: 10.3389/Fimmu.2020.01743 32849621 PMC7417365

[B213] Tracey K. J. (2002). The Inflammatory Reflex. Nature 420, 853. doi: 10.1038/nature01321nature01321[pii 12490958

[B214] Trager U. Tabrizi S. J. (2013). Peripheral Inflammation in Neurodegeneration. J. Mol. Med. 91, 673. doi: 10.1007/s00109-013-1026-0 23546523

[B215] Uriz-Huarte A. Date A. Ang H. Ali S. Brady H. J. M. Fuchter M. J. (2020). The Transcriptional Repressor REV-ERB as a Novel Target for Disease. Bioorg. Med. Chem. Lett. 30, 127395. doi: 10.1016/j.bmcl.2020.127395 32738989

[B216] Videnovic A. Klerman E. B. Wang W. Marconi A. Kuhta T. Zee P. C. (2017). Timed Light Therapy for Sleep and Daytime Sleepiness Associated With Parkinson Disease A Randomized Clinical Trial. JAMA Neurol. 74, 411. doi: 10.1001/jamaneurol.2016.5192 28241159 PMC5470356

[B217] Vijayan V. Pradhan P. Braud L. Fuchs H. R. Gueler F. Motterlini R.. (2019). Human and Murine Macrophages Exhibit Differential Metabolic Responses to Lipopolysaccharide - A Divergent Role for Glycolysis. Redox Biol. 22, 101147. doi: 10.1016/j.redox.2019.101147 30825774 PMC6396203

[B218] Wang G. Z. Hickey S. L. Shi L. Huang H. C. Nakashe P. Koike N.. (2015). Cycling Transcriptional Networks Optimize Energy Utilization on a Genome Scale. Cell Rep. 13, 1868. doi: 10.1016/j.celrep.2015.10.043 26655902 PMC4680985

[B219] Wang X. L. Kooijman S. Gao Y. Tzeplaeff L. Cosquer B. Milanova I.. (2021). Microglia-Specific Knock-Down of Bmal1 Improves Memory and Protects Mice From High Fat Diet-Induced Obesity. Mol. Psychiatry. doi: 10.1038/s41380-021-01169-z PMC8760060 34050326

[B220] Wang S. Li F. Lin Y. K. Wu B. J. (2020). Targeting REV-ERB Alpha for Therapeutic Purposes: Promises and Challenges. Theranostics 10, 4168. doi: 10.7150/thno.43834 32226546 PMC7086371

[B221] Wang L. Pavlou S. Du X. Bhuckory M. Xu H. Chen M. (2019). Glucose Transporter 1 Critically Controls Microglial Activation Through Facilitating Glycolysis. Mol. Neurodegener. 14:2. doi: 10.1186/s13024-019-0305-9 30634998 PMC6329071

[B222] Wang X. L. Wolff S. E. C. Korpel N. Milanova I. Sandu C. Rensen P. C. N.. (2020). Deficiency of the Circadian Clock Gene Bmal1 Reduces Microglial Immunometabolism. Front. Immunol. 11, 586399. doi: 10.3389/Fimmu.2020.586399 33363534 PMC7753637

[B223] Wardlaw S. M. Phan T. X. Saraf A. Chen X. M. Storm D. R. (2014). Genetic Disruption of the Core Circadian Clock Impairs Hippocampus-Dependent Memory. Learn. Memory 21, 417. doi: 10.1101/lm.035451.114 PMC4105720 25034823

[B224] Weber B. Barros L. F. (2015). The Astrocyte: Powerhouse and Recycling Center. Csh Perspect. Biol. 7, a020396. doi: 10.1101/cshperspect.a020396 PMC4665076 25680832

[B225] Weberpals M. Hermes M. Hermann S. Kummer M. P. Terwel D. Semmler A.. (2009). NOS2 Gene Deficiency Protects From Sepsis-Induced Long-Term Cognitive Deficits. J. Neurosci. 29, 14177. doi: 10.1523/Jneurosci.3238-09.2009 19906966 PMC6665081

[B226] Widmann C. N. Heneka M. T. (2014). Long-Term Cerebral Consequences of Sepsis. Lancet Neurol. 13, 630. doi: 10.1016/S1474-4422(14)70017-1 24849863

[B227] Wolff S. E. C. Wang X. L. Jiao H. Sun J. Kalsbeek A. Yi C. X.. (2020). The Effect of Rev-Erbalpha Agonist SR9011 on the Immune Response and Cell Metabolism of Microglia. Front. Immunol. 11, 550145. doi: 10.3389/fimmu.2020.550145 33101272 PMC7546349

[B228] Yamajuku D. Shibata Y. Kitazawa M. Katakura T. Urata H. Kojima T.. (2011). Cellular DBP and E4BP4 Proteins Are Critical for Determining the Period Length of the Circadian Oscillator. FEBS Lett. 585, 2217. doi: 10.1016/j.febslet.2011.05.038 21635892

[B229] Yang G. R. Chen L. H. Grant G. R. Paschos G. Song W. L. Musiek E. S.. (2016). Timing of Expression of the Core Clock Gene Bmal1 Influences Its Effects on Aging and Survival. Sci. Transl. Med. 8 (324), 324ra16. doi: 10.1126/scitranslmed.aad3305 PMC4870001 26843191

[B230] Yang L. K. Guo C. F. Zhu J. Feng Y. Chen W. L. Feng Z. Z.. (2017). Increased Levels of Pro-Inflammatory and Anti-Inflammatory Cellular Responses in Parkinson’s Disease Patients: Search for a Disease Indicator. Med. Sci. Monitor. 23, 2972. doi: 10.12659/Msm.904240 PMC5484607 28624842

[B231] Yang X. O. Pappu B. P. Nurieva R. Akimzhanov A. Kang H. S. Chung Y.. (2008). T Helper 17 Lineage Differentiation Is Programmed by Orphan Nuclear Receptors ROR Alpha and ROR Gamma. Immunity 28, 29. doi: 10.1016/j.immuni.2007.11.016 18164222 PMC2587175

[B232] Yang G. Zhang J. Jiang T. Monslow J. Tang S. Y. Todd L.. (2020). Bmal1 Deletion in Myeloid Cells Attenuates Atherosclerotic Lesion Development and Restrains Abdominal Aortic Aneurysm Formation in Hyperlipidemic Mice. Arterioscler. Thromb. Vasc. Biol. 40, 1523. doi: 10.1161/ATVBAHA.120.314318 32321308 PMC7285859

[B233] Yan L. J. Xiao M. Chen R. Cai Z. (2013). Metabolic Dysfunction of Astrocyte: An Initiating Factor in Beta-Amyloid Pathology? Aging Neurodegener. 1, 7. 24443714 PMC3891850

[B234] Yoo I. D. Park M. W. Cha H. W. Yoon S. Boonpraman N. Yi S. S.. (2020). Elevated CLOCK and BMAL1 Contribute to the Impairment of Aerobic Glycolysis From Astrocytes in Alzheimer’s Disease. Int. J. Mol. Sci. 21, 7862. doi: 10.3390/Ijms21217862 PMC7660350 33114015

[B235] Yu X. F. Rollins D. Ruhn K. A. Stubblefield J. J. Green C. B. Kashiwada M.. (2013). T(H)17 Cell Differentiation Is Regulated by the Circadian Clock. Science 342, 727. doi: 10.1126/science.1243884 24202171 PMC4165400

[B236] Zenaro E. Pietronigro E. Della Bianca V. Piacentino G. Marongiu L. Budui S.. (2015). Neutrophils Promote Alzheimer’s Disease-Like Pathology and Cognitive Decline *via* LFA-1 Integrin. Nat. Med. 21, 880. doi: 10.1038/nm.3913 26214837

[B237] Zhang J. Ke K. F. Liu Z. Qiu Y. H. Peng Y. P. (2013). Th17 Cell-Mediated Neuroinflammation Is Involved in Neurodegeneration of Abeta1-42-Induced Alzheimer’s Disease Model Rats. PloS One 8, e75786. doi: 10.1371/journal.pone.0075786 24124514 PMC3790825

[B238] Zhang R. Lahens N. F. Ballance H. I. Hughes M. E. Hogenesch J. B. (2014). A Circadian Gene Expression Atlas in Mammals: Implications for Biology and Medicine. Proc. Natl. Acad. Sci. U. S. A. 111, 16219. doi: 10.1073/pnas.1408886111 25349387 PMC4234565

[B239] Zhang S. L. Lahens N. F. Yue Z. Arnold D. M. Pakstis P. P. Schwarz J. E.. (2021). A Circadian Clock Regulates Efflux by the Blood-Brain Barrier in Mice and Human Cells. Nat. Commun. 12, 617. doi: 10.1038/s41467-020-20795-9 33504784 PMC7841146

[B240] Zhang E. E. Liu Y. Dentin R. Pongsawakul P. Y. Liu A. C. Hirota T.. (2010). Cryptochrome Mediates Circadian Regulation of cAMP Signaling and Hepatic Gluconeogenesis. Nat. Med. 16, 1152. doi: 10.1038/nm.2214 20852621 PMC2952072

[B241] Zhao Y. F. Qiong Z. Zhang J. F. Lou Z. Y. Zu H. B. Wang Z. G.. (2018). The Synergy of Aging and LPS Exposure in a Mouse Model of Parkinson’s Disease. Aging Dis. 9, 785. doi: 10.14336/Ad.2017.1028 30271656 PMC6147589

